# Unusual sub-genus associations of faecal *Prevotella* and *Bacteroides* with specific dietary patterns

**DOI:** 10.1186/s40168-016-0202-1

**Published:** 2016-10-21

**Authors:** Francesca De Filippis, Nicoletta Pellegrini, Luca Laghi, Marco Gobbetti, Danilo Ercolini

**Affiliations:** 1 Department of Agricultural Sciences, Division of Microbiology, University of Naples Federico II, Via Università 100, 80055 Portici, Italy; 2 Department of Food Science, University of Parma, Parco Area delle Scienze 48/A, 43124 Parma, Italy; 3 Department of Agricultural and Food Sciences, Alma Mater Studiorum University of Bologna, viale Fanin 44, 40127 Bologna, Italy; 4 Inter-Departmental Centre for Industrial Agri-Food Research, Alma Mater Studiorum University of Bologna, Piazza Goidanich 60, 47521 Cesena, Italy; 5 Department of Soil, Plant and Food Science, University of Bari Aldo Moro, Via Amendola 165/a, 70126 Bari, Italy

**Keywords:** Gut microbiota, Oligotyping, Plant-based diet, Omnivore diet

## Abstract

**Background:**

Diet has a recognized effect in shaping gut microbiota. Many studies link an increase in *Prevotella* to high-fibre diet, while *Bacteroides* abundance is usually associated with the consumption of animal fat and protein-rich diets. Nevertheless, closely related species and strains may harbour different genetic pools; therefore, further studies should aim to understand whether species of the same genus are consistently linked to dietary patterns or equally responsive to diet variations. Here, we used oligotyping of 16S rRNA gene sequencing data to exploit the diversity within *Prevotella* and *Bacteroides* genera in faecal samples of omnivore and non-omnivore subjects from a previously studied cohort.

**Results:**

A great heterogeneity was found in oligotype composition. Nevertheless, different oligotypes within the same genus showed distinctive correlation patterns with dietary components and metabolome. We found that some *Prevotella* oligotypes are significantly associated with the plant-based diet but some are associated with animal-based nutrients, and the same applies to *Bacteroides*. Therefore, an indiscriminate association of Bacteroidetes genera with specific dietary patterns may lead to an oversimplified vision that does not take into account sub-genus diversity and the different possible responses to dietary components.

**Conclusions:**

We demonstrated that *Prevotella* and *Bacteroides* oligotypes show distinctive correlation patterns with dietary components and metabolome. These results substantiate a current oversimplification of diet-dependent microbe-host associations and highlighted that sub-genus differences must be taken into account when planning gut microbiota modulation for health benefits

**Electronic supplementary material:**

The online version of this article (doi:10.1186/s40168-016-0202-1) contains supplementary material, which is available to authorized users.

## Background

The important contribution of the gut microbiota to human health and disease is widely recognized [1]. Habitual diet plays a key role in shaping the gut microbial community composition [2–6], and sudden dietary changes are reflected in a prompt response of the gut symbionts [7]. Bacteroidetes are among the most abundant bacterial groups found in the gut, and many genera within this *phylum* are recognized to be diet-responsive; in particular, high-fibre diet is commonly associated with an increase in *Prevotella*, while *Bacteroides* abundance is linked to the consumption of diet rich in animal fat and protein [3–5, 7, 8]. Nevertheless, closely related species and strains may harbour different genetic pools [9–11].

No studies have so far posed the question of whether species of the same genus are consistently linked to dietary patterns or equally responsive to diet variations. The high genetic diversity found between and within *Prevotella* spp. might explain the highly different behaviour observed when considering the genus-level identification [11]. In fact, only some of the subjects reported an increase of this genus after administration of a fibre-rich diet [8], while *P. copri* was supposed to drive chronic inflammation in HIV patients [12] and to induce insulin resistance [13]. Therefore, species and even strain variability have to be considered in order to understand how to modulate the gut microbiota for therapeutic purposes [11]. The current knowledge is limited by the common use of a de novo clustering approach, relying on the clustering of sequences into operational taxonomic units (OTUs) at 97 % of similarity, often resulting in phylogenetically mixed units [14] and leading to taxonomic assignment at the genus level [15]. This approach may fail to resolve ecologically important differences between closely related organisms [16]. On the contrary, even small differences in ribosomal RNA (rRNA) sequence can predict substantial genomic and ecological variation [14, 17]. Oligotyping is an alternative approach, which decomposes a given taxon, or 97 % OTU, into high-resolution units (“oligotypes”) by considering the nucleotide positions identified as the most information-rich and allows resolution at the species level and below [16].

In a recent cohort study, we investigated the associations of microbiota and metabolome with habitual diets, and the OTU-based analyses of the 16S rRNA gene sequences yielded the usual associations of *Prevotella* with plant-based and *Bacteroides* with animal-based diets [2]. Gut *Prevotella* oligotypes were recently supposed to be host-specific and suggested as possible markers useful to track the origin of faecal contamination [18, 19]. Here, we explored the diversity of Bacteroidetes genera in faecal samples of omnivore and non-omnivore subjects of the previously studied cohort [2] using oligotyping of 16S rRNA gene sequencing data and highlight for the first time that heterogeneity within *Prevotella* and *Bacteroides* prevent a comprehensive association of these genera with specific dietary patterns.

## Methods

We used the oligotyping technique to explore differences within *Bacteroides* and *Prevotella* populations in omnivore (*n* = 44) and non-omnivore (self-declared ovo-lacto vegetarians and vegans, *n* = 93) subjects of a previously studied cohort [2]. Subject recruitment, DNA extraction, sequencing of the V1–V3 regions of rRNA gene, and metabolomics were carried out as recently described [2]. Raw reads were quality-filtered as follows: reads were trimmed at the first ambiguous base or when the average quality score dropped below 25 within a 50-bp-long window, and reads shorter than 500 bp and with >1 primer mismatch were discarded. High-quality reads were aligned to the Greengenes alignment template by using PyNAST 1.2.1 [20], and alignment was further trimmed to equal length by using the o-smart-trim script included in the oligotyping package v.1.0 [15]. In order to avoid biases due to different sequencing depths, all samples were rarefied at 4500 reads after raw read quality filtering. Taxonomy assignment of all the reads was carried out by using the RDP classifier [21]. Reads assigned to *Prevotella* or *Bacteroides* genera were extracted, and entropy analysis and oligotyping were carried out separately as described by the developers. High-entropy positions were chosen to compute the oligotypes (-C option): 106, 121, 137, 272, 349, 462, 502, 544, 549, 739, 746, 778, 851 and 98 and 251, 319, 432, 440, 462, 463, 471, 477, 502, 526, 528, 549, 578, 746, 778, 847 and 851 for *Prevotella* and *Bacteroides* dataset, respectively. To minimize the impact of sequencing errors, we required an oligotype to be represented by at least 100 reads (-M option). Moreover, rare oligotypes present in less than 10 samples were discarded (-s option). These parameters led to 61 (omnivores *n* = 17; non-omnivores *n* = 44) and 136 (omnivores *n* = 44; non-omnivores *n* = 92) samples left in *Prevotella* and *Bacteroides* dataset, respectively. BLASTn (match/mismatch scores: 1, −2; gap cost: linear) was used to query the representative sequences against the NCBI nr database, and the top hit was considered for taxonomic assignment. Statistical analyses and plotting were carried out in R environment (https://www.r-project.org). Pairwise Wilcoxon tests were used in order to determine significant differences in specific oligotype abundance according to diets. Pairwise Spearman’s correlations between oligotype relative abundance and diet or metabolome were computed and plotted by using the *psych* and *made4* packages. A cladogram of representative sequences was generated using MrBayes (http://mrbayes.sourceforge.net) and depicted using the Interactive Tree of Life (http://itol.embl.de).

## Results

**Fig. 1:**
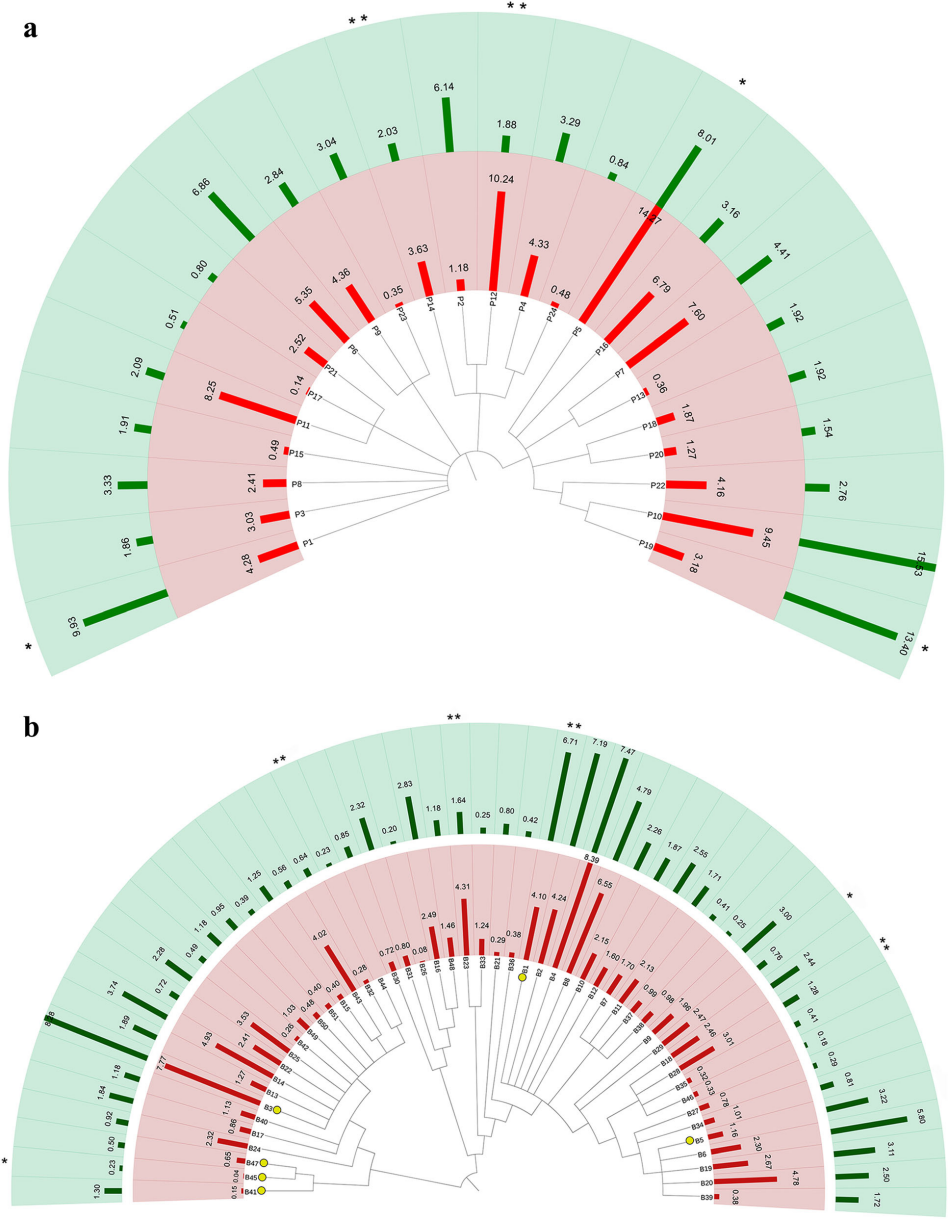
Differences in oligotype composition between omnivores and non-omnivores. Dendrogram of *Prevotella* (**a**) and *Bacteroides* (**b**) oligotype representative sequences and their average relative abundance in omnivores (*inner circle*) and non-omnivores (*outer circle*). *Asterisks* denote oligotypes significantly different in abundance between the two groups (*P < 0.05; **P < 0.01). *Yellow circles* denote oligotypes showing 100 % match with sequences in the NCBI nr database

**Fig. 2:**
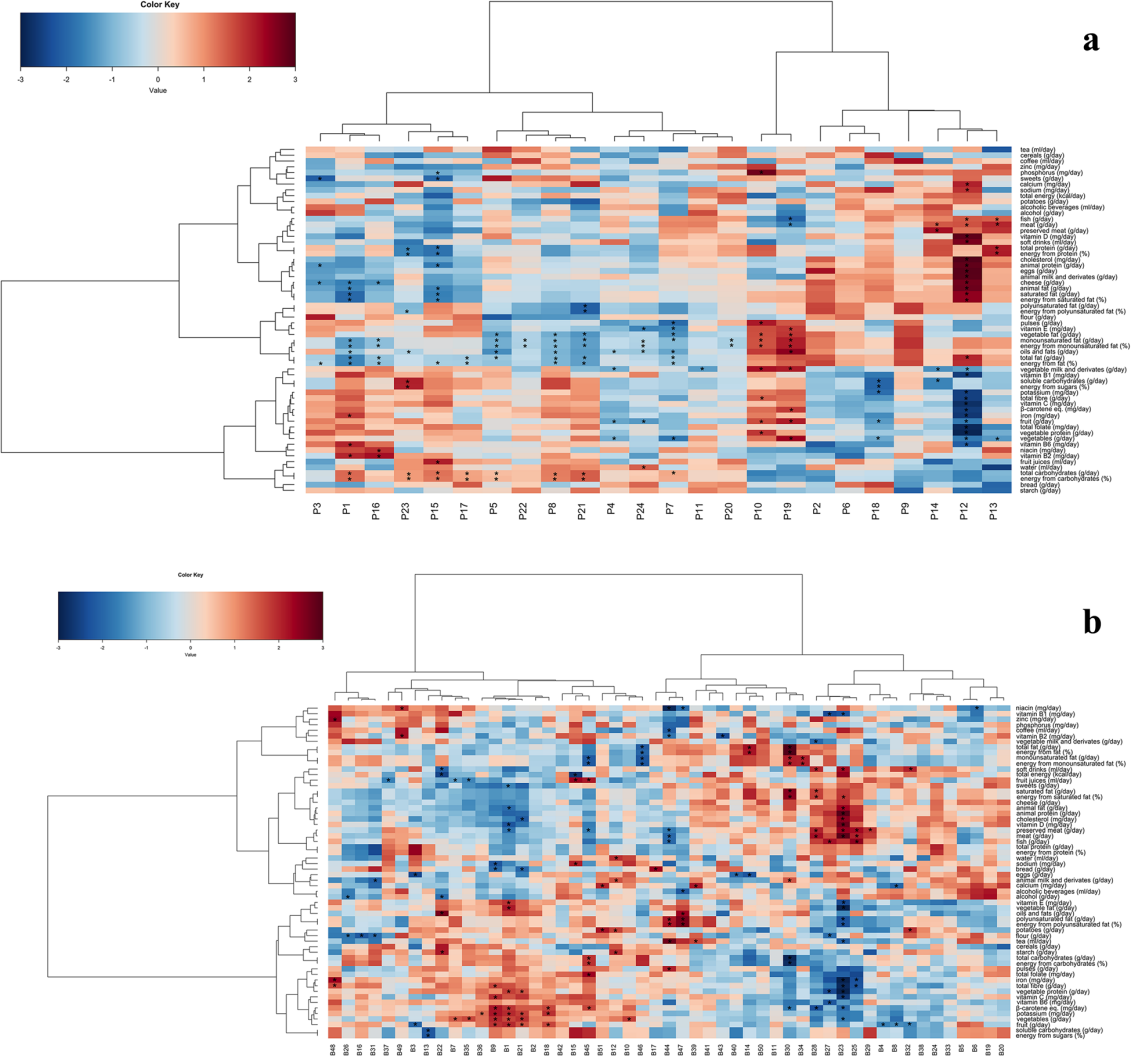
Correlation patterns between oligotypes and diet. Correlation between *Prevotella* (**a**) and *Bacteroides* (**b**) oligotypes and dietary data. Heatplot showing Spearman’s correlations between oligotypes and dietary data. *Rows* and *columns* are clustered by Euclidean distance and Ward linkage hierarchical clustering. The intensity of the *colours* represents the degree of association between oligotypes and foods/nutrients as measured by Spearman’s correlations. *Asterisks* denote significant correlations after *P* value corrections (*P* < 0.05)

The relative abundance of *Prevotella* and *Bacteroides* ranged from 86.7 to 0.08 % and from 58 to 0.13 %, respectively, regardless of the habitual diet. Since no significant differences in *Prevotella* and *Bacteroides* oligotype profiles between vegans and vegetarians were detected, the two vegetable-based diets were grouped together (as non-omnivore). A total of 24 and 51 oligotypes were identified for *Prevotella* and *Bacteroides*, respectively, but the number of different oligotypes detected was not related to the relative abundance of the genus in the faecal samples (Additional file 1: Figure S1). Oligotype representative sequences and BLASTn results are reported in Additional file 2: Table S1. High inter- and intra-individual diversity was found, with some diet-specific signatures (Additional file 3: Figure S2). *Prevotella* oligotypes P5, P11, P12 and P14 were clearly more abundant in omnivores, while P1, P10 and P19 prevailed in non-omnivores (Fig. 1a). Consistently, P12 showed positive correlations with animal-origin nutrients and foodstuffs (Fig. 2a) and with urinary trimethylamine oxide (TMAO) concentration (Additional file 4: Figure S3A). On the contrary, P1, P10 and P19 were strongly correlated with vegetable-based diets (Fig. 2a) and to faecal short-chain fatty acid (SCFA) levels (Additional file 4: Figure S3A). Besides showing a similar correlation pattern, P10 and P19 had higher sequence similarity, differing from all the others (Additional file 5: Figure S4A); they were identified as *P. loescheii* by best BLASTn match. All the other *Prevotella* oligotypes matched *P. copri* sequences, although none with 100 % identity (Additional file 5: Figure S4A and Additional file 2: Table S1A). A higher number of oligotypes was found within *Bacteroides* (Additional file 3: Figure S2B and Additional file 2: Table S1B), and 10 different species were identified, some showing exact matches with sequences in the nr database (Additional file 5: Figure S4B and Additional file 2: Table S1B). *Bacteroides* oligotype B1, B2 and B6 had higher abundance in non-omnivores, and B44 was found only in non-omnivores. On the contrary, B23, B27, B28 and B29 prevailed in omnivores (Fig. 1b); showed positive correlations with animal fats, proteins and animal-origin foodstuffs (Fig. 2b); and consistently with urinary TMAO (Additional file 4: Figure S3B). In particular, B29 was associated with fish, meat and preserved meat consumption, while B27 was specifically associated with fish. Finally, the co-occurrence/exclusion pattern highlights that some oligotypes frequently co-occur, e.g. *Prevotella* oligotypes P10 and P19 (*P* < 0.05), and are mutually exclusive with P5, P11 and P12, associated with an omnivore diet (Additional file 6: Figure S5).

## Discussion

Gut microbiota is a complex *consortium* of microbial species and strains [9]. However, analyses based on OTU clustering methodology often result in genus-level classification and may underestimate this diversity [16]. This oversimplified vision does not take into account the possible different responses that species or strains belonging to the same genus may have to dietary components [8, 10]. The oligotyping approach was proposed as a powerful tool allowing resolution at the species level and below. It was successfully used to explain the microbial complexity of the oral microbiota [15] and to associate diversity within *Blautia* and *Prevotella* genera to different animal hosts [13, 18]. Moreover, differentiations in faecal *Bacteroides* oligotype composition were previously observed in Western and agrarian populations [14]. Here, we oligotyped *Bacteroides* and *Prevotella* 16S rRNA gene sequences in human faecal samples and demonstrated that specific oligotypes within these genera may be associated with the habitual diet. Consistently, oligotypes associated with an omnivore diet were positively correlated to TMAO levels, a metabolite derived from carnitine and choline catabolism and related to the development of cardiovascular diseases [22]. On the contrary, those linked to a vegetable-based diet showed positive correlations with SCFA, derived from carbohydrate fermentation and associated with beneficial outcomes for the host, such as anti-inflammatory and anti-carcinogenic effects [23, 24]. Our results highlighted how an indiscriminate association of a whole genus with a specific dietary pattern may lead to an oversimplified vision of the correlations between gut microbiota and diet, which does not take into account the diversity existing within the same genus or even within the same species [9, 11] and the possible different responses of these variants to dietary components [8, 10].

## Conclusions

Sub-genus-level diversity can play a key role in establishing the interconnection between gut microbiome, diet, microbial metabolome and host response, and this should be taken into account in diet-based intervention studies aiming at changing gut microbiota structure and functions in order to gather specific health benefits. The consistency of the correlative patterns between oligotypes, diet and metabolome suggests the intriguing presence of a diet-driven adaptive selection, associated with different metabolic and enzymatic profiles. Different *Prevotella* and *Bacteroides* oligotypes, although identified as the same species, showed differential relative abundance in omnivores and non-omnivores and a consistent correlation pattern with foodstuffs/nutrients and metabolome, highlighting a possible distinct behaviour below the genus level never demonstrated before and the presence of different putative strains with diverse metabolic potential, possibly selected by the habitual diet.

## Additional files

Additional file 1: Figure S1. Bar plot of *Prevotella* (A) and *Bacteroides* (B) genera relative abundance ordered by size and line chart showing the number of different oligotypes identified in the same subject. (TIF 7240 kb )
Additional file 2: Table S1A.
*Prevotella* oligotypes representative sequences and BLASTn top hit. **Table S1B.** *Bacteroides* oligotypes representative sequences and BLASTn top hit. (DOCX 129 kb)
Additional file 3: Figure S2. Pie charts showing the average relative abundance of *Prevotella* (A) and *Bacteroides* (B) oligotypes in omnivore (left side) and non-omnivore (right side) subjects. For clarity, only *Bacteroides* oligotypes showing >1 % abundance in at least 10 % of the subjects in each group are shown. (TIF 9270 kb)
Additional file 4: Figure S3. Correlation between *Prevotella* (A) and *Bacteroides* (B) oligotypes and metabolome. Heatplot showing Spearman’s correlations between oligotypes, urinary TMAO and faecal SCFA levels. Rows and columns are clustered by Euclidean distance and Ward linkage hierarchical clustering. The intensity of the colours represents the degree of association between oligotypes and metabolites as measured by Spearman’s correlations. Asterisks denote significant correlations after *P* value corrections (*P* < 0.05). (TIF 8860 kb)
Additional file 5: Figure S4. Heatplot showing the percent nucleotide identity between each pair of oligotypes within *Prevotella* (A) and *Bacteroides* (B) genera. Each oligotype is identified with the best match found in the NCBI nr database, with the percent of query coverage and the percent of identity. Row and columns are clustered by Horn distance and Ward linkage hierarchical clustering. (TIF 8620 kb)
Additional file 6: Figure S5. Significant co-occurrence and co-exclusion relationships between *Prevotella* (A) and *Bacteroides* (B) oligotypes. Strong correlations are indicated by large circles, whereas weak correlations are indicated by small circles. The colours of the scale bar denote the nature of the correlation, with 1 indicating a perfectly positive correlation (dark blue) and −1 indicating a perfectly negative correlation (dark red) between two oligotypes. Only significant correlations (*P* < 0.05) are shown. (TIF 8840 kb)
