# Supplementary figures and images for: Unusual sub-genus associations of faecal Prevotella and Bacteroides with specific dietary patterns
Source: Microbiome. 2016 Oct 21;4:57. doi: 10.1186/s40168-016-0202-1 (PMC5073871; doi:10.1186/s40168-016-0202-1)

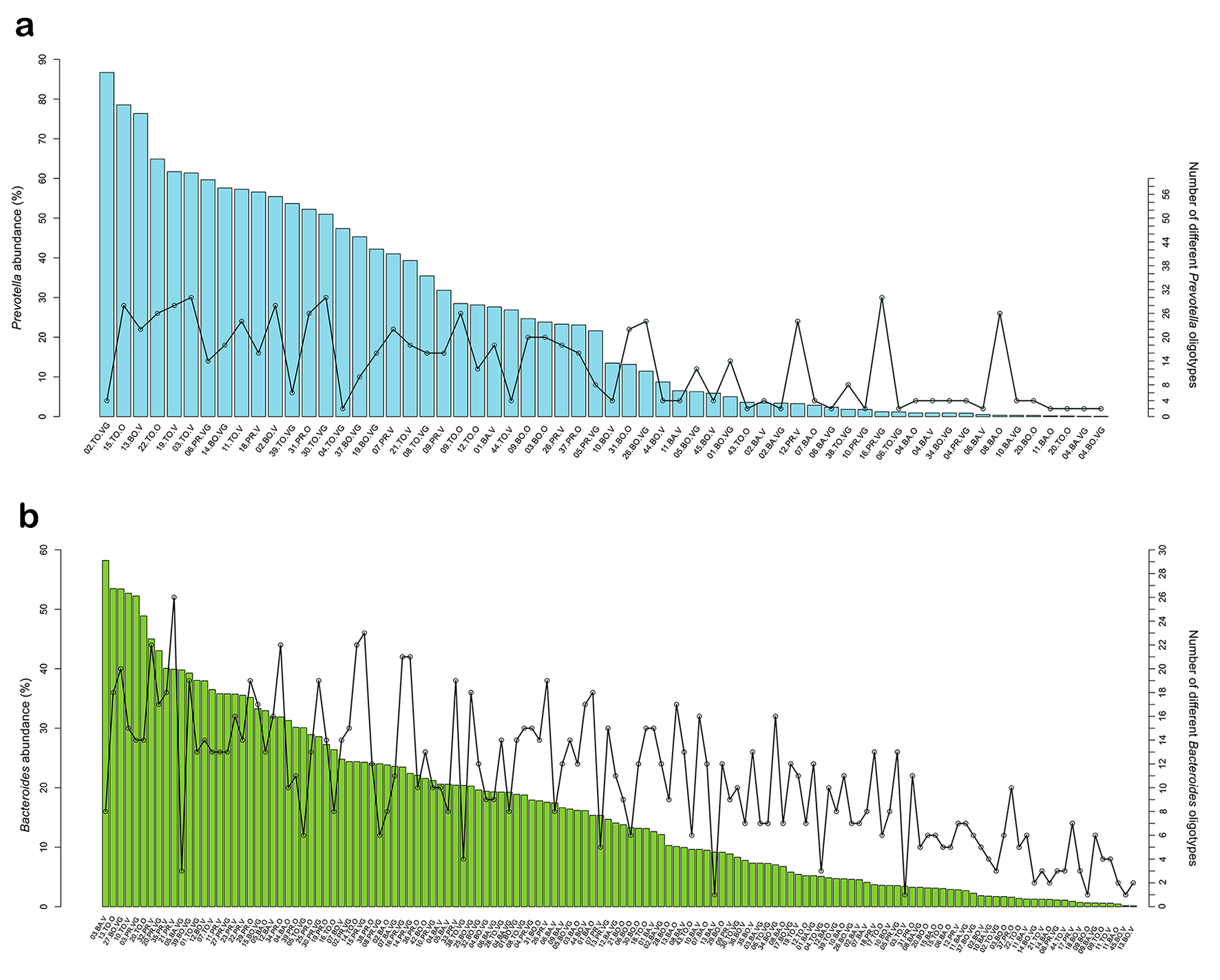

Supplement: Additional file 1: Figure S1. — Bar plot of Prevotella (A) and Bacteroides (B) genera relative abundance ordered by size and line chart showing the number of different oligotypes identified in the same subject. (TIF 7240 kb ) [file 40168_2016_202_MOESM1_ESM.tif]

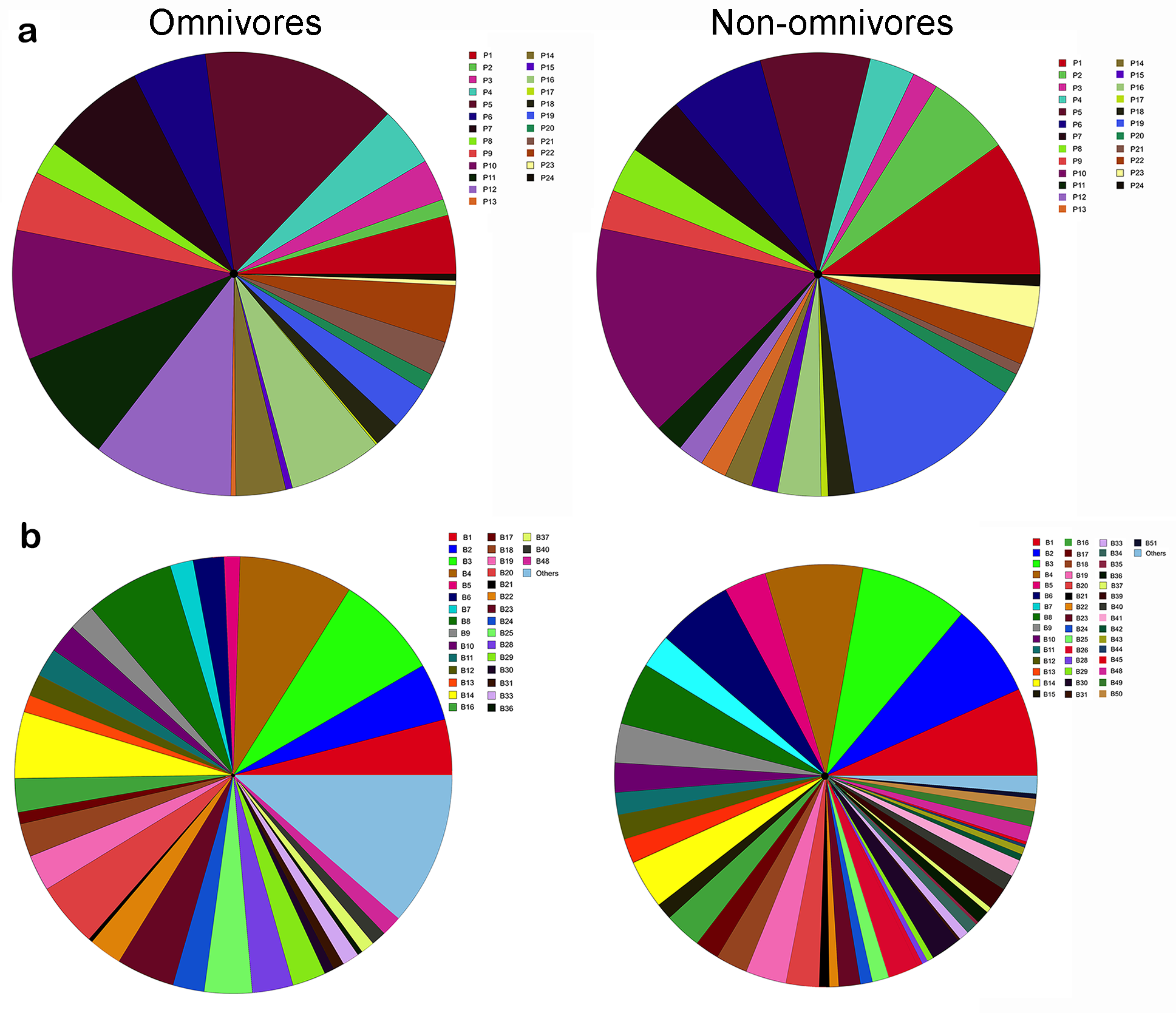

Supplement: Additional file 3: Figure S2. — Pie charts showing the average relative abundance of Prevotella (A) and Bacteroides (B) oligotypes in omnivore (left side) and non-omnivore (right side) subjects. For clarity, only Bacteroides oligotypes showing >1 % abundance in at least 10 % of the subjects in each group are shown. (TIF 9270 kb) [file 40168_2016_202_MOESM3_ESM.tif]

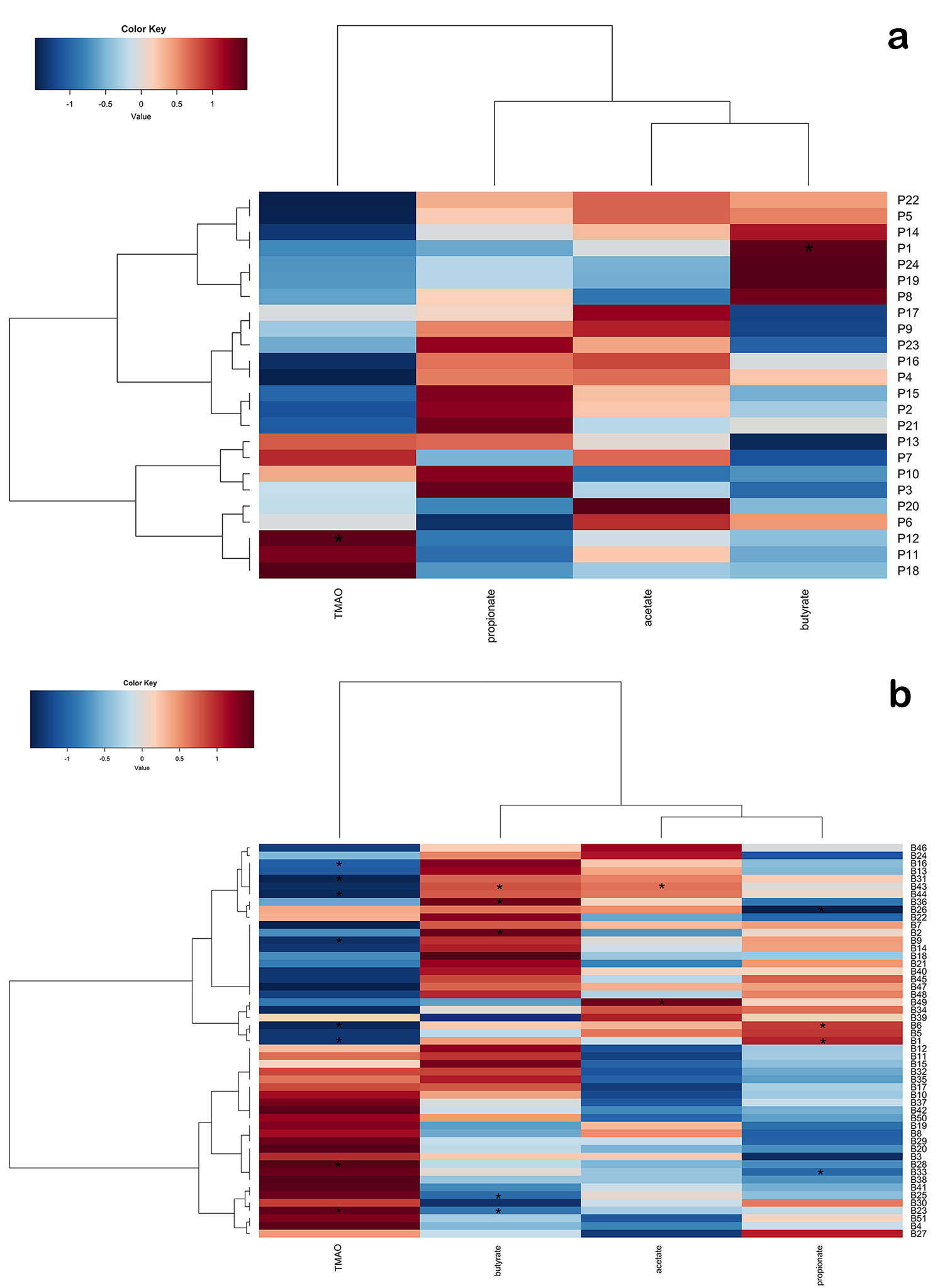

Supplement: Additional file 4: Figure S3. — Correlation between Prevotella (A) and Bacteroides (B) oligotypes and metabolome. Heatplot showing Spearman’s correlations between oligotypes, urinary TMAO and faecal SCFA levels. Rows and columns are clustered by Euclidean distance and Ward linkage hierarchical clustering. The intensity of the colours represents the degree of association between oligotypes and metabolites as measured by Spearman’s correlations. Asterisks denote significant correlations after P value corrections (P < 0.05). (TIF 8860 kb) [file 40168_2016_202_MOESM4_ESM.tif]

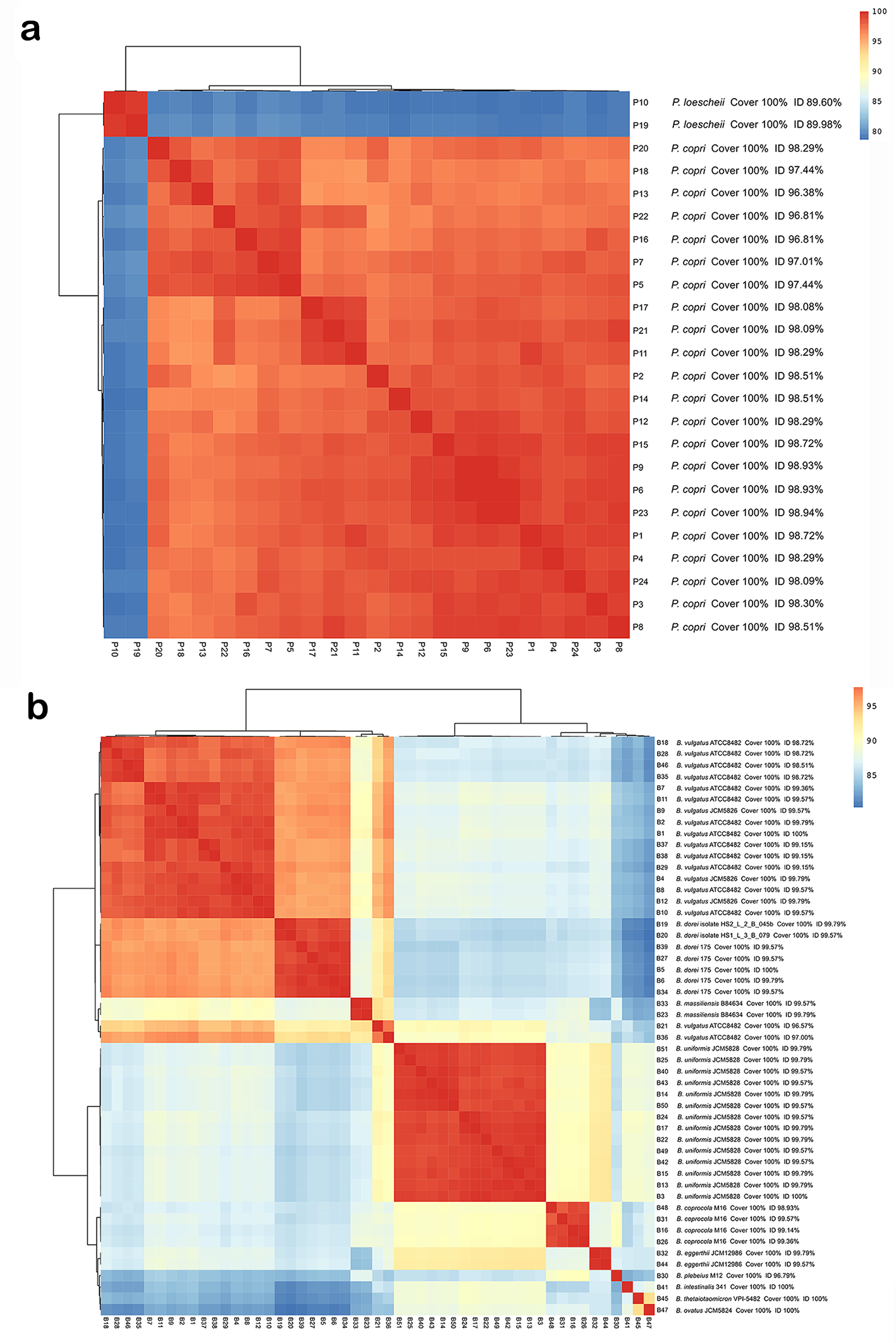

Supplement: Additional file 5: Figure S4. — Heatplot showing the percent nucleotide identity between each pair of oligotypes within Prevotella (A) and Bacteroides (B) genera. Each oligotype is identified with the best match found in the NCBI nr database, with the percent of query coverage and the percent of identity. Row and columns are clustered by Horn distance and Ward linkage hierarchical clustering. (TIF 8620 kb) [file 40168_2016_202_MOESM5_ESM.tif]

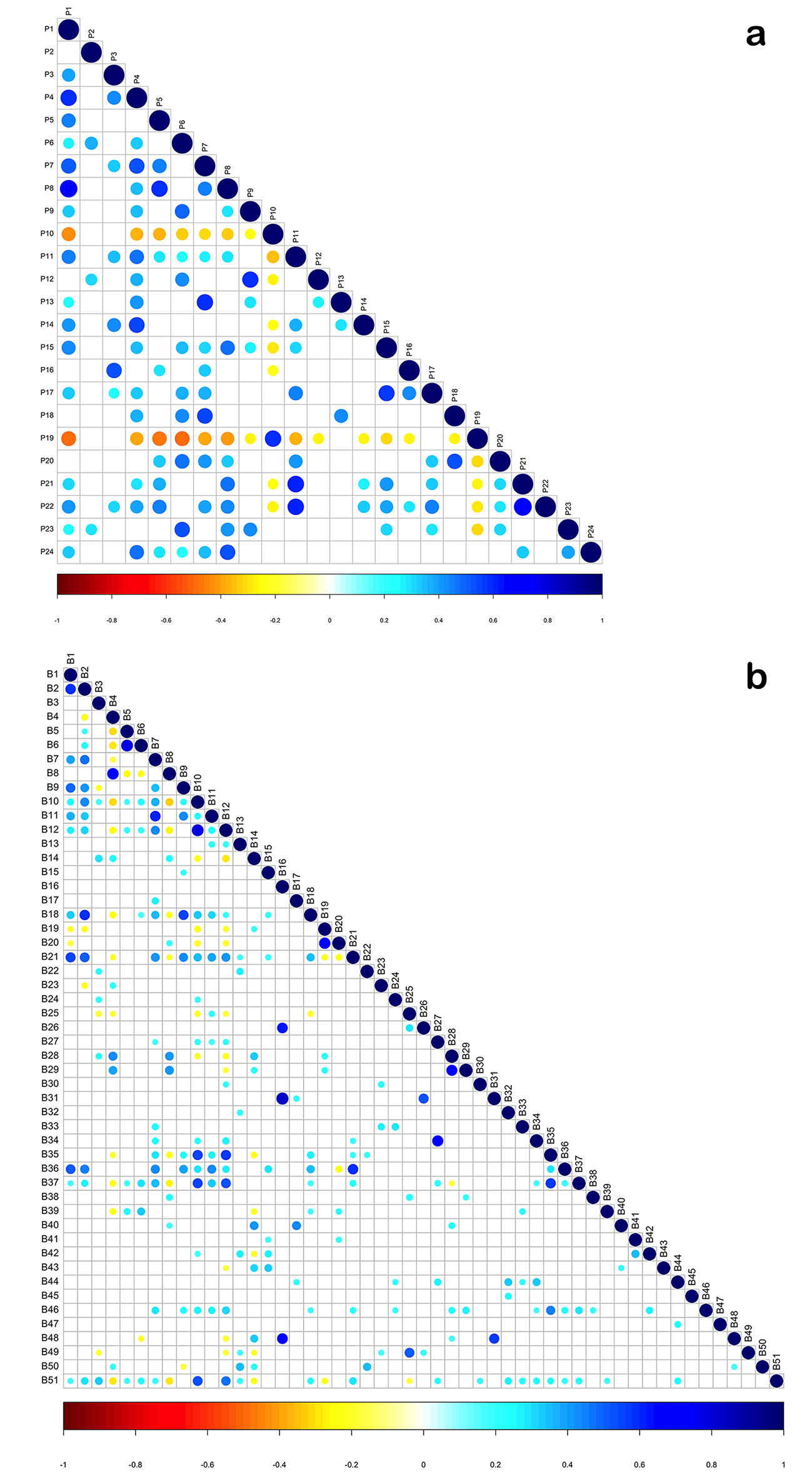

Supplement: Additional file 6: Figure S5. — Significant co-occurrence and co-exclusion relationships between Prevotella (A) and Bacteroides (B) oligotypes. Strong correlations are indicated by large circles, whereas weak correlations are indicated by small circles. The colours of the scale bar denote the nature of the correlation, with 1 indicating a perfectly positive correlation (dark blue) and −1 indicating a perfectly negative correlation (dark red) between two oligotypes. Only significant correlations (P < 0.05) are shown. (TIF 8840 kb) [file 40168_2016_202_MOESM6_ESM.tif]
