# Supplementary material for: Unusual sub-genus associations of faecal Prevotella and Bacteroides with specific dietary patterns
Source: Microbiome. 2016 Oct 21;4:57. doi: 10.1186/s40168-016-0202-1 (PMC5073871; doi:10.1186/s40168-016-0202-1)
Supplement: Additional file 2: Table S1A. — Prevotella oligotypes representative sequences and BLASTn top hit. Table S1B. Bacteroides oligotypes representative sequences and BLASTn top hit. (DOCX 129 kb) [file 40168_2016_202_MOESM2_ESM.docx]

**Table S1A.** *Prevotella* oligotypes representative sequences and BLASTn top-hit.

| **Oligotype ID** | **Representative Sequence** | **BLASTn top-hit** |
| --- | --- | --- |
| **P1** | ACGCTAGCTACAGGCTTAACACATGCAAGTCGAGGGGAAACGACATCGAAAGCTTGCTTTTGATGGGCGTCGACCGGCGCACGGGTGAGTAACGCGTATCCAACCTGCCCACCACTTGGGGATAACCTTGCGAAAGTAAGACTAATACCCAATGATATCTCTAGAAGACATCTGAAAGAGATTAAAGATTTATCGGTGATGGATGGGGATGCGTCTGATTAGCTTGTTGGCGGGGTAACGGCCCACCAAGGCAACGATCAGTAGGGGTTCTGAGAGGAAGGTCCCCCACATTGGAACTGAGACACGGTCCAAACTCCTACGGGAGGCAGCAGTGAGGAATATTGGTCAATGGACGAGAGTCTGAACCAGCCAAGTAGCGTGCAGGAAGACGGCCCTATGGGTTGTAAACTGCTTTTATAAGGGAATAAAGTGAGTCTCGTGAGACTTTTTGCATGTACCTTATGAATAA | *Prevotella copri* JCM 13464 |
| **P2** | ACGCTAGCTACAGGCTTAACACATGCAAGTCGAGGGGAAACGACATCGAAAGCTTGCTTTTGATGGGCGTCGACCGGCGCACGGGTGAGTAACGCGTATCCAACCTGCCCACCACTTGGGGATAACCTTGCGAAAGTAAGACTAATACCCAATGACGTCTCTAGAAGACATCTGAAGGAGATTAAAGATTTATCGGTGATGGATGGGGATGCGTCTGATTAGCTTGTTGGCGGGGTAACGGCCCACCAAGGCGACGATCAGTAGGGGTTCTGAGAGGAAGGTCCCCCACATTGGAACTGAGACACGGTCCAAACTCCTACGGGAGGCAGCAGTGAGGAATATTGGTCAATGGGCGAGAGCCTGAACCAGCCAAGTAGCGTGCAGGATGACGGCCCTATGGGTTGTAAACTGCTTTTATAAGGGAATAAAGTGAGTCTCGTGAGACTTTTTGCATGTACCTTATGAATAA | *Prevotella copri* CB7 |
| **P3** | ACGCTAGCTACAGGCTTAACACATGCAAGTCGAGGGGAAACGACATCGAAAGCTTGCTTTTGATGGGCGTCGACCGGCGCACGGGTGAGTAACGCGTATCCAACCTGCCCACCACTTGGGGATAACCTTGCGAAAGTAAGACTAATACCCAATGATATCTCTAGAAGACATCTGAAAGAGATTAAAGATTTATCGGTGATGGATGGGGATGCGTCTGATTAGCTTGTTGGCGGGGTAACGGCCCACCAAGGCAACGATCAGTAGGGGTTCTGAGAGGAAGGTCCCCCCACATTGGAACTGAGACACGGTCCAAACTCCTACGGGAGGCAGCAGTGAGGAATATTGGTCAATGGACGAGAGTCTGAACCAGCCAAGTAGCGTGCAGGAAGACGGCCCTATGGGTTGTAAACTGCTTTTATAAGGGAATAAAGTTAGTCTCGTGAGACTTTTTGCATGTACCTTATGAATAA | *Prevotella copri* JCM 13464 |
| **P4** | ACGCTAGCTACAGGCTTAACACATGCAAGTCGAGGGGAAACGACATCGAAAGCTTGCTTTTGATGGGCGTCGACCGGCGCACGGGTGAGTAACGCGTATCCAACCTGCCCACCACTTGGGGATAACCTTGCGAAAGTAAGACTAATACCCAATGACGTCTCTAGAAGACATCTGAAAGAGATTAAAGATTTATCGGTGATGGATGGGGATGCGTCTGATTAGCTTGTTGGCGGGGTAACGGCCCACCAAGGCAACGATCAGTAGGGGTTCTGAGAGGAAGGTCCCCCACATTGGAACTGAGACACGGTCCAAACTCCTACGGGAGGCAGCAGTGAGGAATATTGGTCAATGGACGAGAGTCTGAACCAGCCAAGTAGCGTGCAGGAAGACGGCCCTATGGGTTGTAAACTGCTTTTATAAGGGAATAAAGTGAGTCTCGTGAGACTTTTTGCATGTACCTTATGAATAA | *Prevotella copri* JCM 13464 |
| **P5** | ACGCTAGCTACAGGCTTAACACATGCAAGTCGAGGGGAAACGATATTGGAAGCTTGCTTCCGATAGGCGTCGACCGGCGCACGGGTGAGTAACGCGTATCCAACCTGCCCACCACTTGGGGATAACCTTGCGAAAGTAAGACTAATACCCAATGATATCTCTAGAAGACATCTGAAAGAGATTAAAGATTTATCGGTGATGGATGGGGATGCGTCTGATTAGCTTGTTGGCGGGGTAACGGCCCACCAAGGCAACGATCAGTAGGGGTTCTGAGAGGAAGGTCCCCCACATTGGAACTGAGACACGGTCCAAACTCCTACGGGAGGCAGCAGTGAGGAATATTGGTCAATGGACGAGAGTCTGAACCAGCCAAGTAGCGTGCAGGAAGACGGCCCTATGGGTTGTAAACTGCTTTTATAAGGGAATAAAGTGAGTCTCGTGAGACTTTTTGCATGTACCTTATGAATAA | *Prevotella copri* JCM 13464 |
| **P6** | ACGCTAGCTACAGGCTTAACACATGCAAGTCGAGGGGAAACGACATCGAAAGCTTGCTTTTGATGGGCGTCGACCGGCGCACGGGTGAGTAACGCGTATCCAACCTGCCCACCACTTGGGGATAACCTTGCGAAAGTAAGACTAATACCCAATGATATCTCTAGAAGACATCTGAAAGAGATTAAAGATTTATCGGTGATGGATGGGGATGCGTCTGATTAGCTTGTTGGCGGGGTAACGGCCCACCAAGGCGACGATCAGTAGGGGTTCTGAGAGGAAGGTCCCCCACATTGGAACTGAGACACGGTCCAAACTCCTACGGGAGGCAGCAGTGAGGAATATTGGTCAATGGACGAGAGTCTGAACCAGCCAAGTAGCGTGCAGGATGACGGCCCTATGGGTTGTAAACTGCTTTTATAAGGGAATAAAGTGAGTCTCGTGAGACTTTTTGCATGTACCTTATGAATAA | *Prevotella copri* CB7 |
| **P7** | ACGCTAGCTACAGGCTTAACACATGCAAGTCGAGGGGAAACGATATTGGAAGCTTGCTTCCGATAGGCGTCGACCGGCGCACGGGTGAGTAACGCGTATCCAACCTGCCCACCACTTGGGGATAACCTTGCGAAAGTAAGACTAATACCCAATGACGTCTCTAGAAGACATCTGAAAGAGATTAAAGATTTATCGGTGATGGATGGGGATGCGTCTGATTAGCTTGTTGGCGGGGTAACGGCCCACCAAGGCAACGATCAGTAGGGGTTCTGAGAGGAAGGTCCCCCACATTGGAACTGAGACACGGTCCAAACTCCTACGGGAGGCAGCAGTGAGGAATATTGGTCAATGGACGAGAGTCTGAACCAGCCAAGTAGCGTGCAGGAAGACGGCCCTATGGGTTGTAAACTGCTTTTATAAGGGAATAAAGTGAGTCTCGTGAGACTTTTTGCATGTACCTTATGAATAA | *Prevotella copri* JCM 13464 |
| **P8** | ACGCTAGCTACAGGCTTAACACATGCAAGTCGAGGGGAAACGACATCGAAAGCTTGCTTTTGATGGGCGTCGACCGGCGCACGGGTGAGTAACGCGTATCCAACCTGCCCACCACTTGGGGATAACCTTGCGAAAGTAAGACTAATACCCAATGATATCTCTAGAAGACATCTGAAAGAGATTAAAGATTTATCGGTGATGGATGGGGATGCGTCTGATTAGCTTGTTGGCGGGGTAACGGCCCACCAAGGCAACGATCAGTAGGGGTTCTGAGAGGAAGGTCCCCCACATTGGAACTGAGACACGGTCCAAACTCCTACGGGGAGGCAGCAGTGAGGAATATTGGTCAATGGACGAGAGTCTGAACCAGCCAAGTAGCGTGCAGGAAGACGGCCCTATGGGTTGTAAACTGCTTTTATAAGGGAATAAAGTGAGTCTCGTGAGACTTTTTGCATGTACCTTATGAATAA | *Prevotella copri* JCM 13464 |
| **P9** | ACGCTAGCTACAGGCTTAACACATGCAAGTCGAGGGGAAACGACATCGAAAGCTTGCTTTTGATGGGCGTCGACCGGCGCACGGGTGAGTAACGCGTATCCAACCTGCCCATCACTTGGGGATAACCTTGCGAAAGTAAGACTAATACCCAATGATATCTCTAGAAGACATCTGAAAGAGATTAAAGATTTATCGGTGATGGATGGGGATGCGTCTGATTAGCTTGTTGGCGGGGTAACGGCCCACCAAGGCGACGATCAGTAGGGGTTCTGAGAGGAAGGTCCCCCACATTGGAACTGAGACACGGTCCAAACTCCTACGGGAGGCAGCAGTGAGGAATATTGGTCAATGGACGAGAGTCTGAACCAGCCAAGTAGCGTGCAGGATGACGGCCCTATGGGTTGTAAACTGCTTTTATAAGGGAATAAAGTGAGTCTCGTGAGACTTTTTGCATGTACCTTATGAATAA | *Prevotella copri* CB7 |
| **P10** | ACGCTAGCTACAGGCTTAACACATGCAAGTCGCGGGGCAGCATGAAGGATGCTTGCATCCTTTGATGGCGACCGGCGCACGGGTGAGTAACGCGTATCCAACCTGCCTGTTACCACGGCACAGCCCGTCGAAAGGCGGATTAATGCCGTATGTGGTCCTTTGCGGGCATCTAATTAGGACTAAAGGTTTAGCGGTAACGGATGGGGATGCGTCCGATTAGCTAGACGGCGGGGTAACGGCCCACCGTGGCTACGATCGGTAGGGGTTCTGAGAGGAAGGTCCCCCACACTGGAACTGAGACACGGTCCAGACTCCTACGGGGAGGCAGCAGTGAGGAATATTGGTCAATGGGCGGAAGCCTGAACCAGCCAAGTAGCGTGAGGGAAGACTGCCCTATGGGTTGTAAACCTCTTTTTGTGCGGGGATAAAGTGAGGGACGTGTCCCTTATTGCAGGTACCGCACGAATAA | *Prevotella loescheii* JCM 8530 |
| **P11** | ACGCTAGCTACAGGCTTAACACATGCAAGTCGAGGGGAAACGACATCGAAAGCTTGCTTTTGATGGGCGTCGACCGGCGCACGGGTGAGTAACGCGTATCCAACCTGCCCACCACTTGGGGATAACCTTGCGAAAGTAAGACTAATACCCAATGATATCTCTAGAAGACATCTGAAAGAGATTAAAGATTTATCGGTGATGGATGGGGATGCGTCTGATTAGCTTGTTGGCGGGGTAACGGCCCACCAAGGCAACGATCAGTAGGGGTTCTGAGAGGAAGGTCCCCCACATTGGAACTGAGACACGGTCCAAACTCCTACGGGAGGCAGCAGTGAGGAATATTGGTCAATGGACGAGAGTCTGAACCAGCCAAGTAGCGTGCAGGAAGACGGCCCTATGGGTTGTAAACTGCTTTTATAAGGGAATAAAGTGAGAGTCGTGACTCTTTTTGCATGTACCTTATGAATAA | *Prevotella copri* JCM 13464 |
| **P12** | ACGCTAGCTACAGGCTTAACACATGCAAGTCGAGGGGAAACGACATCGAAAGCTTGCTTTTGATGGGCGTCGACCGGCGCACGGGTGAGTAACGCGTATCCAACCTGCCCATCACTTGGGGATAACCTTGCGAAAGTAAGACTAATACCCAATGACGTCTCTAGAAGACATCTGAAAGAGATTAAAGATTTATCGGTGATGGATGGGGATGCGTCTGATTAGCTTGTTGGCGGGGTAACGGCCCACCAAGGCGACGATCAGTAGGGGTTCTGAGAGGAAGGTCCCCACATTGGAACTGAGACACGGTCCAAACTCCTACGGGAGGCAGCAGTGAGGAATATTGGTCAATGGACGAGAGTCTGAACCAGCCAAGTAGCGTGCAGGATGACGGCCCTATGGGTTGTAAACTGCTTTTATAAGGGAATAAAGTGAGTCTCGTGAGACTTTTTGCATGTACCTTATGAATAA | *Prevotella copri* CB7 |
| **P13** | ACGCTAGCTACAGGCTTAACACATGCAAGTCGAGGGGAAACGATATTGGAAGCTTGCTTCCGATAGGCGTCGACCGGCGCACGGGTGAGTAACGCGTATCCAACCTGCCCATCACTTGGGGATAACCTTGCGAAAGTAAGACTAATACCCAATGACGTCTCTAGAAGACATCTGAAAGAGATTAAAGATTTATCGGTGATGGATGGGGATGCGTCTGATTAGCTTGTTGGCGGGGTAACGGCCCACCAAGGCAACGATCAGTAGGGGTTCTGAGAGGAAGGTCCCCACATTGGAACTGAGACACGGTCCAAACTCCTACGGGAGGCAGCAGTGAGGAATATTGGTCAATGGACGAGAGTCTGAACCAGCCAAGTAGCGTGCAGGAAGACGGCCCTATGGGTTGTAAACTGCTTTTATAAGGGAATAAAGTTAGTCTCGTGAGACTTTTTGCATGTACCTTATGAATAA | *Prevotella copri* CB7 |
| **P14** | ACGCTAGCTACAGGCTTAACACATGCAAGTCGAGGGGAAACGACATCGAAAGCTTGCTTTTGATGGGCGTCGACCGGCGCACGGGTGAGTAACGCGTATCCAACCTGCCCACCACTTGGGGATAACCTTGCGAAAGTAAGACTAATACCCAATGACGTCTCTAGAAGACATCTGAAAGAGATTAAAGATTTATCGGTGATGGATGGGGATGCGTCTGATTAGCTTGTTGGCGGGGTAATGGCCCACCAAGGCAACGATCAGTAGGGGTTCTGAGAGGAAGGTCCCCCCACATTGGAACTGAGACACGGTCCAAACTCCTACGGGAGGCAGCAGTGAGGAATATTGGTCAATGGACGAGAGTCTGAACCAGCCAAGTAGCGTGCAGGATGACGGCCCTATGGGTTGTAAACTGCTTTTATAAGGGAATAAAGTGAGCCTCGTGAGGCTTTTTGCATGTACCTTATGAATAA | *Prevotella copri* JCM 13464 |
| **P15** | ACGCTAGCTACAGGCTTAACACATGCAAGTCGAGGGGAAACGACATCGAAAGCTTGCTTTTGATGGGCGTCGACCGGCGCACGGGTGAGTAACGCGTATCCAACCTGCCCACCACTTGGGGATAACCTTGCGAAAGTAAGACTAATACCCAATGATATCTCTAGAAGGCATCTGAAAGAGATTAAAGATTTATCGGTGATGGATGGGGATGCGTCTGATTAGCTTGTTGGCGGGGTAACGGCCCACCAAGGCAACGATCAGTAGGGGTTCTGAGAGGAAGGTCCCCCACATTGGAACTGAGACACGGTCCAAACTCCTACGGGAGGCAGCAGTGAGGAATATTGGTCAATGGACGAGAGTCTGAACCAGCCAAGTAGCGTGCAGGATGACGGCCCTATGGGTTGTAAACTGCTTTTATAAGGGAATAAAGTGAGTCTCGTGAGACTTTTTGCATGTACCTTATGAATAA | *Prevotella copri* JCM 13464 |
| **P16** | ACGCTAGCTACAGGCTTAACACATGCAAGTCGAGGGGAAACGATATTGGAAGCTTGCTTCCGATAGGCGTCGACCGGCGCACGGGTGAGTAACGCGTATCCAACCTGCCCACCACTTGGGGATAACCTTGCGAAAGTAAGACTAATACCCAATGATATCTCTAGAAGACATCTGAAAGAGATTAAAGATTCATCGGTGATGGATGGGGATGCGTCTGATTAGCTTGTTGGCGGGGTAACGGCCCACCAAGGCAACGATCAGTAGGGGTTCTGAGAGGAAGGTCCCCCCACATTGGAACTGAGACACGGTCCAAACTCCTACGGGAGGCAGCAGTGAGGAATATTGGTCAATGGACGAGAGTCTGAACCAGCCAAGTAGCGTGCAGGAAGACGGCCCTATGGGTTGTAAACTGCTTTTATAAGGGAATAAAGTTAGTCTCGTGAGACTTTTTGCATGTACCTTATGAATAA | *Prevotella copri* JCM 13464 |
| **P17** | ACGCTAGCTACAGGCTTAACACATGCAAGTCGAGGGGAAACGACATCGAAAGCTTGCTTTTGATGGGCGTCGACCGGCGCACGGGTGAGTAACGCGTATCCAACCTGCCCACCACTTGGGGATAACCTTGCGAAAGTAAGACTAATACCCAATGATATCTCTAGAAGACATCTGAAAGAGATTAAAGATTTATCGGTGATGGATGGGGATGCGTCTGATTAGCTTGTTGGCGGGGTAACGGCCCACCAAGGCGACGATCAGTAGGGGTTCTGAGAGGAAGGTCCCCCACATTGGAACTGAGACACGGTCCAAACTCCTACGGGAGGCAGCAGTGAGGAATATTGGTCAATGGACGAGAGTCTGAACCAGCCAAGTAGCGTGTAGGAAGACGGCCCTATGGGTTGTAAACTGCTTTTATAAGGGAATAAAGTGGGAGTCGTGACTCTTTTTGCATGTACCTTATGAATAA | *Prevotella copri* JCM 13464 |
| **P18** | ACGCTAGCTACAGGCTTAACACATGCAAGTCGAGGGGAAACGATATTGGAAGCTTGCTTCCGATAGGCGTCGACCGGCGCACGGGTGAGTAACGCGTATCCAACCTGCCCACCACTTGGGGATAACCTTGCGAAAGTAAGACTAATACCCAATGACGTCTCTAGAAGACATCTGAAAGAGATTAAAGATTTATCGGTGATGGATGGGGATGCGTCTGATTAGCTTGTTGGCGGGGTAACGGCCCACCAAGGCGACGATCAGTAGGGGTTCTGAGAGGAAGGTCCCCACATTGGAACTGAGACACGGTCCAAACTCCTACGGGAGGCAGCAGTGAGGAATATTGGTCAATGGGCGAGAGCCTGAACCAGCCAAGTAGCGTGCAGGAAGACGGCCCTATGGGTTGTAAACTGCTTTTATAAGGGAATAAAGTGAGTCTCGTGAGACTTTTTGCATGTACCTTATGAATAA | *Prevotella copri* JCM 13464 |
| **P19** | ACGCTAGCTACAGGCTTAACACATGCAAGTCGCGGGGCAGCATGAAGGATGCTTGCATCCTTTGATGGCGACCGGCGCACGGGTGAGTAACGCGTATCCAACCTGCCTGTTACCACGGCACAGCCCGTCGAAAGGCGGATTAATGCCGTATGTGGTCCTTTGCGGGCATCTAATTAGGACTAAAGGTTTAGCGGTAACGGATGGGGATGCGTCCGATTAGCTAGACGGCGGGGTAACGGCCCACCGTGGCTACGATCGGTAGGGGTTCTGAGAGGAAGGTCCCCCACACTGGAACTGAGACACGGTCCAGACTCCTACGGGAGGCAGCAGTGAGGAATATTGGTCAATGGGCGGAAGCCTGAACCAGCCAAGTAGCGTGAGGGAAGACTGCCCTATGGGTTGTAAACCTCTTTTGTGCGGGGATAAAGTGAGGGACGTGTCCCTTATTGCAGGTACCGCACGAATAA | *Prevotella loescheii* JCM 8530 |
| **P20** | ACGCTAGCTACAGGCTTAACACATGCAAGTCGAGGGGAAACGATATTGGAAGCTTGCTTCCGATAGGCGTCGACCGGCGCACGGGTGAGTAACGCGTATCCAACCTGCCCACCACTTGGGGATAACCTTGCGAAAGTAAGACTAATACCCAATGATATCTCTAGAAGACATCTGAAAGAGATTAAAGATTTATCGGTGATGGATGGGGATGCGTCTGATTAGCTTGTTGGCGGGGTAACGGCCCACCAAGGCGACGATCAGTAGGGGTTCTGAGAGGAAGGTCCCCCACATTGGAACTGAGACACGGTCCAAACTCCTACGGGAGGCAGCAGTGAGGAATATTGGTCAATGGGCGAGAGCCTGAACCAGCCAAGTAGCGTGCAGGATGACGGCCCTATGGGTTGTAAACTGCTTTTATAAGGGAATAAAGTGAGTCTCGTGAGACTTTTTGCATGTACCTTATGAATAA | *Prevotella copri* JCM 13464 |
| **P21** | ACGCTAGCTACAGGCTTAACACATGCAAGTCGAGGGGAAACGACATCGAAAGCTTGCTTTTGATGGGCGTCGACCGGCGCACGGGTGAGTAACGCGTATCCAACCTGCCCACCACTTGGGGATAACCTTGCGAAAGTAAGACTAATACCCAATGATATCTCTAGAAGACATCTGAAAGAGATTAAAGATTTATCGGTGATGGATGGGGATGCGTCTGATTAGCTTGTTGGCGGGGTAACGGCCCACCAAGGCAACGATCAGTAGGGGTTCTGAGAGGAAGGTCCCCCACATTGGAACTGAGACACGGTCCAAACTCCTACGGGGAGGCAGCAGTGAGGAATATTGGTCAATGGACGAGAGTCTGAACCAGCCAAGTAGCGTGCAGGAAGACGGCCCTATGGGTTGTAAACTGCTTTTATAAGGGAATAAAGTGAGAGTCGTGACTCTTTTTGCATGTACCTTATGAATAA | *Prevotella copri* JCM 13464 |
| **P22** | ACGCTAGCTACAGGCTTAACACATGCAAGTCGAGGGGAAACGATATTGGAAGCTTGCTTCCGATAGGCGTCGACCGGCGCACGGGTGAGTAACGCGTATCCAACCTGCCCACCACTTGGGGATAACCTTGCGAAAGTAAGACTAATACCCAATGATATCTCTAGAAGACATCTGAAAGAGATTAAAGATTTATCGGTGATGGATGGGGATGCGTCTGATTAGCTTGTTGGCGGGGTAACGGCCCACCAAGGCAACGATCAGTAGGGGTTCTGAGAGGAAGGTCCCCCACATTGGAACTGAGACACGGTCCAAACTCCTACGGGGAGGCAGCAGTGAGGAATATTGGTCAATGGACGAGAGTCTGAACCAGCCAAGTAGCGTGCAGGAAGACGGCCCTATGGGTTGTAAACTGCTTTTATAAGGGAATAAAGTGAGAGTCGTGACTCTTTTTGCATGTACCTTATGAATAA | *Prevotella copri* JCM 13464 |
| **P23** | ACGCTAGCTACAGGCTTAACACATGCAAGTCGAGGGGAAACGACATCGAAAGCTTGCTTTTGATGGGCGTCGACCGGCGCACGGGTGAGTAACGCGTATCCAACCTGCCCACCACTTGGGGATAACCTTGCGAAAGTAAGACTAATACCCAATGATATCTCTAGAAGACATCTGAAAGAGATTAAAGATTTATCGGTGATGGATGGGGATGCGTCTGATTAGCTTGTTGGCGGGGTAACGGCCCACCAAGGCGACGATCAGTAGGGGTTCTGAGAGGAAGGTCCCCCACATTGGAACTGAGACACGGTCCAAACTCCTACGGGGAGGCAGCAGTGAGGAATATTGGTCAATGGACGAGAGTCTGAACCAGCCAAGTAGCGTGCAGGATGACGGCCCTATGGGTTGTAAACTGCTTTTATAAGGGAATAAAGTGAGTCTCGTGAGACTTTTTGCATGTACCTTATGAATAA | *Prevotella copri* JCM 13464 |
| **P24** | ACGCTAGCTACAGGCTTAACACATGCAAGTCGAGGGGAAACGACATCGAAAGCTTGCTTTTGATGGGCGTCGACCGGCGCACGGGTGAGTAACGCGTATCCAACCTGCCCACCACTTGGGGATAACCTTGCGAAAGTAAGACTAATACCCAATGACGTCTCTAGAAGACATCTGAAAGAGATTAAAGATTTATCGGTGATGGATGGGGATGCGTCTGATTAGCTTGTTGGCGGGGTAACGGCCCACCAAGGCAACGATCAGTAGGGGTTCTGAGAGGAAGGTCCCCCACATTGGAACTGAGACACGGTCCAAACTCCTACGGGGAGGCAGCAGTGAGGAATATTGGTCAATGGACGAGAGTCTGAACCAGCCAAGTAGCGTGCAGGAAGACGGCCCTATGGGTTGTAAACTGCTTTTATAAGGGAATAAAGTGAGTCTCGTGAGACTTTTTGCATGTACCTTATGAATAA | *Prevotella copri* JCM 13464 |

**Table S1B.** *Bacteroides* oligotypes representative sequences and BLASTn top-hit.

| **Oligotype ID** | **Representative Sequence** | **BLASTn top-hit** |
| --- | --- | --- |
| **B1** | ACGCTAGCTACAGGCTTAACACATGCAAGTCGAGGGGCAGCATGGTCTTAGCTTGCTAAGGCCGATGGCGACCGGCGCACGGGTGAGTAACACGTATCCAACCTGCCGTCTACTCTTGGACAGCCTTCTGAAAGGAAGATTAATACAAGATGGCATCATGAGTCCGCATGTTCACATGATTAAAGGTATTCCGGTAGACGATGGGGATGCGTTCCATTAGATAGTAGGCGGGGTAACGGCCCACCTAGTCTTCGATGGATAGGGGTTCTGAGAGGAAGGTCCCCCACATTGGAACTGAGACACGGTCCAAACTCCTACGGGAGGCAGCAGTGAGGAATATTGGTCAATGGGCGAGAGCCTGAACCAGCCAAGTAGCGTGAAGGATGACTGCCCTATGGGTTGTAAACTTCTTTTATAAAGGAATAAAGTCGGGTATGGATACCCGTTTGCATGTACTTTATGAATAA | *Bacteroides vulgatus* ATCC 8482 |
| **B2** | ACGCTAGCTACAGGCTTAACACATGCAAGTCGAGGGGCAGCATGGTCTTAGCTTGCTAAGGCCGATGGCGACCGGCGCACGGGTGAGTAACACGTATCCAACCTGCCGTCTACTCTTGGACAGCCTTCTGAAAGGAAGATTAATACAAGATGGCATCATGAGTCCGCATGTTCACATGATTAAAGGTATTCCGGTAGACGATGGGGATGCGTTCCATTAGATAGTAGGCGGGGTAACGGCCCACCTAGTCTTCGATGGATAGGGGTTCTGAGAGGAAGGTCCCCCACATTGGAACTGAGACACGGTCCAAACTCCTACGGGAGGCAGCAGTGAGGAATATTGGTCAATGGGCGAGAGCCTGAACCAGCCAAGTAGCGTGAAGGATGACTGCCCTATGGGTTGTAAACTTCTTTTATAAAGGATAAAGTCGGGTATGGATACCCGTTTGCATGTACTTTATGAATAA | *Bacteroides vulgatus* ATCC 8482 |
| **B3** | ACGCTAGCTACAGGCTTAACACATGCAAGTCGAGGGGCAGCATGAACTTAGCTTGCTAAGTTTGATGGCGACCGGCGCACGGGTGAGTAACACGTATCCAACCTGCCGATGACTCGGGGATAGCCTTTCGAAAGAAAGATTAATACCCGATGGCATAGTTCTTCCGCATGGTAGAACTATTAAAGAATTTCGGTCATCGATGGGGATGCGTTCCATTAGGTTGTTGGCGGGGTAACGGCCCACCAAGCCTTCGATGGATAGGGGTTCTGAGAGGAAGGTCCCCCACATTGGAACTGAGACACGGTCCAAACTCCTACGGGAGGCAGCAGTGAGGAATATTGGTCAATGGACGAGAGTCTGAACCAGCCAAGTAGCGTGAAGGATGACTGCCCTATGGGTTGTAAACTTCTTTTATACGGGAATAAAGTGAGGCACGTGTGCCTTTTTGTATGTACCGTATGAATAA | *Bacteroides uniformis* JCM 5828 |
| **B4** | ACGCTAGCTACAGGCTTAACACATGCAAGTCGAGGGGCAGCATGGTCTTAGCTTGCTAAGGCCGATGGCGACCGGCGCACGGGTGAGTAACACGTATCCAACCTGCCGTCTACTCTTGGACAGCCTTCTGAAAGGAAGATTAATACAAGATGGCATCATGAGTCCGCATGTTCACATGATTAAAGGTATTCCGGTAGACGATGGGGATGCGTTCCATTAGATAGTAGGCGGGGTAACGGCCCACCTAGTCTTCGATGGATAGGGGTTCTGAGAGGAAGGTCCCCCACATTGGAACTGAGACACGGTCCAAACTCCTACGGGGAGGCAGCAGTGAGGAATATTGGTCAATGGGCGAGAGCCTGAACCAGCCAAGTAGCGTGAAGGATGACTGCCCTATGGGTTGTAAACTTCTTTTATAAAGGAATAAAGTCGGGTATGGATACCCGTTTGCATGTACTTTATGAATAA | *Bacteroides vulgatus* JCM 5826 |
| **B5** | ACGCTAGCTACAGGCTTAACACATGCAAGTCGAGGGGCAGCATGGTCTTAGCTTGCTAAGGCTGATGGCGACCGGCGCACGGGTGAGTAACACGTATCCAACCTGCCGTCTACTCTTGGCCAGCCTTCTGAAAGGAAGATTAATCCAGGATGGGATCATGAGTTCACATGTCCGCATGATTAAAGGTATTTTCCGGTAGACGATGGGGATGCGTTCCATTAGATAGTAGGCGGGGTAACGGCCCACCTAGTCAACGATGGATAGGGGTTCTGAGAGGAAGGTCCCCCACATTGGAACTGAGACACGGTCCAAACTCCTACGGGAGGCAGCAGTGAGGAATATTGGTCAATGGGCGATGGCCTGAACCAGCCAAGTAGCGTGAAGGATGACTGCCCTATGGGTTGTAAACTTCTTTTATAAAGGAATAAAGTCGGGTATGCATACCCGTTTGCATGTACTTTATGAATAA | *Bacteroides dorei* 175 |
| **B6** | ACGCTAGCTACAGGCTTAACACATGCAAGTCGAGGGGCAGCATGGTCTTAGCTTGCTAAGGCTGATGGCGACCGGCGCACGGGTGAGTAACACGTATCCAACCTGCCGTCTACTCTTGGCCAGCCTTCTGAAAGGAAGATTAATCCAGGATGGGATCATGAGTTCACATGTCCGCATGATTAAAGGTATTTTCCGGTAGACGATGGGGATGCGTTCCATTAGATAGTAGGCGGGGTAACGGCCCACCTAGTCAACGATGGATAGGGGTTCTGAGAGGAAGGTCCCCCACATTGGAACTGAGACACGGTCCAAACTCCTACGGGAGGCAGCAGTGAGGAATATTGGTCAATGGGCGATGGCCTGAACCAGCCAAGTAGCGTGAAGGATGACTGCCCTATGGGTTGTAAACTTCTTTTATAAAGGATAAAGTCGGGTATGCATACCCGTTTGCATGTACTTTATGAATAA | *Bacteroides dorei* 175 |
| **B7** | ACGCTAGCTACAGGCTTAACACATGCAAGTCGAGGGGCAGCATGGTCTTAGCTTGCTAAGACTGATGGCGACCGGCGCACGGGTGAGTAACACGTATCCAACCTGCCGTCTACTCTTGGACAGCCTTCTGAAAGGAAGATTAATACAAGATGGCATCATGAGTCCGCATGTTCACATGATTAAAGGTATTCCGGTAGACGATGGGGATGCGTTCCATTAGATAGTAGGCGGGGTAACGGCCCACCTAGTCTTCGATGGATAGGGGTTCTGAGAGGAAGGTCCCCCACATTGGAACTGAGACACGGTCCAAACTCCTACGGGAGGCAGCAGTGAGGAATATTGGTCAATGGGCGAGAGCCTGAACCAGCCAAGTAGCGTGAAGGATGACTGCCCTATGGGTTGTAAACTTCTTTTATAAAGGATAAAGTCGGGTATGGATACCCGTTTGCATGTACTTTATGAATAA | *Bacteroides vulgatus* ATTC 8482 |
| **B8** | ACGCTAGCTACAGGCTTAACACATGCAAGTCGAGGGGCAGCATGGTCTTAGCTTGCTAAGGCCGATGGCGACCGGCGCACGGGTGAGTAACACGTATCCAACCTGCCGTCTACTCTTGGACAGCCTTCTGAAAGGAAGATTAATACAAGATGGCATCATGAGTCCGCATGTTCACATGATTAAAGGTATTCCGGTAGACGATGGGGATGCGTTCCATTAGATAGTAGGCGGGGTAACGGCCCACCTAGTCTTCGATGGATAGGGGTTCTGAGAGGAAGGTCCCCCACATTGGAACTGAGACACGGTCCAAACTCCTACGGGGAGGCAGCAGTGAGGAATATTGGTCAATGGGCGAGAGCCTGAACCAGCCAAGTAGCGTGAAGGATGACTGCCCTATGGGTTGTAAACTTCTTTTATAAAGGATAAAGTCGGGTATGGATACCCGTTTGCATGTACTTTATGAATAA | *Bacteroides vulgatus* ATTC 8482 |
| **B9** | ACGCTAGCTACAGGCTTAACACATGCAAGTCGAGGGGCAGCATGGTCTTAGCTTGCTAAGGCCGATGGCGACCGGCGCACGGGTGAGTAACACGTATCCAACCTGCCGTCTACTCTTGGACAGCCTTCTGAAAGGAAGATTAATACAAGATGGCATCATGAGTCCACATGTTCACATGATTAAAGGTATTCCGGTAGACGATGGGGATGCGTTCCATTAGATAGTAGGCGGGGTAACGGCCCACCTAGTCTTCGATGGATAGGGGTTCTGAGAGGAAGGTCCCCCACATTGGAACTGAGACACGGTCCAAACTCCTACGGGAGGCAGCAGTGAGGAATATTGGTCAATGGGCGAGAGCCTGAACCAGCCAAGTAGCGTGAAGGATGACTGCCCTATGGGTTGTAAACTTCTTTTATAAAGGAATAAAGTCGGGTATGCATACCCGTTTGCATGTACTTTATGAATAA | *Bacteroides vulgatus* JCM 5826 |
| **B10** | ACGCTAGCTACAGGCTTAACACATGCAAGTCGAGGGGCAGCATGGTCTTAGCTTGCTAAGGCCGATGGCGACCGGCGCACGGGTGAGTAACACGTATCCAACCTGCCGTCTACTCTTGGACAGCCTTCTGAAAGGAAGATTAATACAAGATGGCATCATGAGTCCGCATGTTCACATGATTAAAGGTATTCCGGTAGACGATGGGGATGCGTTCCATTAGATAGTAGGCGGGGTAACGGCCCACCTAGTCTTCGATGGATAGGGGTTCTGAGAGGAAGGTCCCCCCACATTGGAACTGAGACACGGTCCAAACTCCTACGGGAGGCAGCAGTGAGGAATATTGGTCAATGGGCGAGAGCCTGAACCAGCCAAGTAGCGTGAAGGATGACTGCCCTATGGGTTGTAAACTTCTTTTATAAAGGATAAAGTCGGGTATGGATACCCGTTTGCATGTACTTTATGAATAA | *Bacteroides vulgatus* ATTC 8482 |
| **B11** | ACGCTAGCTACAGGCTTAACACATGCAAGTCGAGGGGCAGCATGGTCTTAGCTTGCTAAGACTGATGGCGACCGGCGCACGGGTGAGTAACACGTATCCAACCTGCCGTCTACTCTTGGACAGCCTTCTGAAAGGAAGATTAATACAAGATGGCATCATGAGTCCGCATGTTCACATGATTAAAGGTATTCCGGTAGACGATGGGGATGCGTTCCATTAGATAGTAGGCGGGGTAACGGCCCACCTAGTCTTCGATGGATAGGGGTTCTGAGAGGAAGGTCCCCCACATTGGAACTGAGACACGGTCCAAACTCCTACGGGAGGCAGCAGTGAGGAATATTGGTCAATGGGCGAGAGCCTGAACCAGCCAAGTAGCGTGAAGGATGACTGCCCTATGGGTTGTAAACTTCTTTTATAAAGGAATAAAGTCGGGTATGGATACCCGTTTGCATGTACTTTATGAATAA | *Bacteroides vulgatus* ATTC 8482 |
| **B12** | ACGCTAGCTACAGGCTTAACACATGCAAGTCGAGGGGCAGCATGGTCTTAGCTTGCTAAGGCCGATGGCGACCGGCGCACGGGTGAGTAACACGTATCCAACCTGCCGTCTACTCTTGGACAGCCTTCTGAAAGGAAGATTAATACAAGATGGCATCATGAGTCCGCATGTTCACATGATTAAAGGTATTCCGGTAGACGATGGGGATGCGTTCCATTAGATAGTAGGCGGGGTAACGGCCCACCTAGTCTTCGATGGATAGGGGTTCTGAGAGGAAGGTCCCCCCACATTGGAACTGAGACACGGTCCAAACTCCTACGGGAGGCAGCAGTGAGGAATATTGGTCAATGGGCGAGAGCCTGAACCAGCCAAGTAGCGTGAAGGATGACTGCCCTATGGGTTGTAAACTTCTTTTATAAAGGAATAAAGTCGGGTATGGATACCCGTTTGCATGTACTTTATGAATAA | *Bacteroides vulgatus* ATTC 8482 |
| **B13** | ACGCTAGCTACAGGCTTAACACATGCAAGTCGAGGGGCAGCATGAACTTAGCTTGCTAAGTTTGATGGCGACCGGCGCACGGGTGAGTAACACGTATCCAACCTGCCGATGACTCGGGGATAGCCTTTCGAAAGAAAGATTAATACCCGATGGCATAGTTCTTCCGCATGGTGGAACTATTAAAGAATTTCGGTCATCGATGGGGATGCGTTCCATTAGGTTGTTGGCGGGGTAACGGCCCACCAAGCCTTCGATGGATAGGGGTTCTGAGAGGAAGGTCCCCCACATTGGAACTGAGACACGGTCCAAACTCCTACGGGAGGCAGCAGTGAGGAATATTGGTCAATGGACGAGAGTCTGAACCAGCCAAGTAGCGTGAAGGATGACTGCCCTATGGGTTGTAAACTTCTTTTATACGGGAATAAAGTGAGGCACGTGTGCCTTTTTGTATGTACCGTATGAATAA | *Bacteroides uniformis* JCM 5828 |
| **B14** | ACGCTAGCTACAGGCTTAACACATGCAAGTCGAGGGGCAGCATGAACTTAGCTTGCTAAGTTTGATGGCGACCGGCGCACGGGTGAGTAACACGTATCCAACCTGCCGATGACTCGGGGATAGCCTTTCGAAAGAAAGATTAATACCCGATGGCATAGTTCTTCCGCATGGTAGAACTATTAAAGAATTTCGGTCATCGATGGGGATGCGTTCCATTAGGTTGTTGGCGGGGTAACGGCCCACCAAGCCTTCGATGGATAGGGGTTCTGAGAGGAAGGTCCCCCACATTGGAACTGAGACACGGTCCAAACTCCTACGGGGAGGCAGCAGTGAGGAATATTGGTCAATGGACGAGAGTCTGAACCAGCCAAGTAGCGTGAAGGATGACTGCCCTATGGGTTGTAAACTTCTTTTATACGGGAATAAAGTGAGGCACGTGTGCCTTTTTGTATGTACCGTATGAATAA | *Bacteroides uniformis* JCM 5828 |
| **B15** | ACGCTAGCTACAGGCTTAACACATGCAAGTCGAGGGGCAGCATGAACTTAGCTTGCTAAGTTTGATGGCGACCGGCGCACGGGTGAGTAACACGTATCCAACCTGCCGATGACTCGGGGATAGCCTTTCGAAAGAAAGATTAATACCCGATGGCATAGTTCTTCCGCATGGTAGAACTATTAAAGAATTTCGGTCATCGATGGGGATGCGTTCCATTAGGTTGTTGGCGGGGTAACGGCCCACCAAGCCTTCGATGGATAGGGGTTCTGAGAGGAAGGTCCCCCACATTGGAACTGAGACACGGTCCAAACTCCTACGGGAGGCAGCAGTGAGGAATATTGGTCAATGGACGAGAGTCTGAACCAGCCAAGTAGCGTGAAGGATGACTGCCCTATGGGTTGTAAACTTCTTTTATACGGGAATAAAGTGAGGCACGCGTGCCTTTTTGTATGTACCGTATGAATAA | *Bacteroides uniformis* JCM 5828 |
| **B16** | ACGCTAGCTACAGGCTTAACACATGCAAGTCGAGGGGCAGCATGAACTTAGCTTGCTAAGTTTGATGGCGACCGGCGCACGGGTGAGTAACACGTATCCAACCTTCCGTTTACTCAGGGATAGCCTTTCGAAAGAAAGATTAATACCTGATAGTATGGTGAGATTGCATGATAGCACCATTAAAGATTTATTGGTAAACGATGGGGATGCGTTCCATTAGGTAGTAGGCGGGGTAACGGCCCACCTAGCCGACGATGGATAGGGGTTCTGAGAGGAAGGTCCCCCACATTGGAACTGAGACACGGTCCAAACTCCTACGGGAGGCAGCAGTGAGGAATATTGGTCAATGGGCGAGAGCCTGAACCAGCCAAGTAGCGTGAAGGATGAAGGTCCTACGGATTGTAAACTTCTTTTATACGGGAATAAAGTATCCTACGTGTAGGATTTTGTATGTACCGTATGAATAA | *Bacteroides coprocola* M16 |
| **B17** | ACGCTAGCTACAGGCTTAACACATGCAAGTCGAGGGGCAGCATGAACTTAGCTTGCTAAGTTTGATGGCGACCGGCGCACGGGTGAGTAACACGTATCCAACCTGCCGATGACTCGGGGATAGCCTTTCGAAAGAAAGATTAATACCCGATGGCATAATTCTTCCGCATGGTAGAACTATTAAAGAATTTCGGTCATCGATGGGGATGCGTTCCATTAGGTTGTTGGCGGGGTAACGGCCCACCAAGCCTTCGATGGATAGGGGTTCTGAGAGGAAGGTCCCCCACATTGGAACTGAGACACGGTCCAAACTCCTACGGGAGGCAGCAGTGAGGAATATTGGTCAATGGACGAGAGTCTGAACCAGCCAAGTAGCGTGAAGGATGACTGCCCTATGGGTTGTAAACTTCTTTTATACGGGAATAAAGTGAGGCACGTGTGCCTTTTTGTATGTACCGTATGAATAA | *Bacteroides uniformis* JCM 5828 |
| **B18** | ACGCTAGCTACAGGCTTAACACATGCAAGTCGAGGGGCAGCATGGTCTTAGCTTGCTAAGGCCGATGGCGACCGGCGCACGGGTGAGTAACACGTATCCAACCTGCCGTCTACTCTTGGACAGCCTTCTGAAAGGAAGATTAATACAAGATGGCATCATGAGTTCACATGTTCACATGATTAAAGGTATTCCGGTAGACGATGGGGATGCGTTCCATTAGATAGTAGGCGGGGTAACGGCCCACCTAGTCTTCGATGGATAGGGGTTCTGAGAGGAAGGTCCCCCACATTGGAACTGAGACACGGTCCAAACTCCTACGGGAGGCAGCAGTGAGGAATATTGGTCAATGGGCGCAGGCCTGAACCAGCCAAGTAGCGTGAAGGATGACTGCCCTATGGGTTGTAAACTTCTTTTATAAAGGATAAAGTCGGGTATGTATACCCGTTTGCATGTACTTTATGAATAA | *Bacteroides vulgatus* ATTC 8482 |
| **B19** | ACGCTAGCTACAGGCTTAACACATGCAAGTCGAGGGGCAGCATGGTCTTAGCTTGCTAAGGCTGATGGCGACCGGCGCACGGGTGAGTAACACGTATCCAACCTGCCGTCTACTCTTGGCCAGCCTTCTGAAAGGAAGATTAATCCAGGATGGGATCATGAGTTCACATGTCCGCATGATTAAAGGTATTTTCCGGTAGACGATGGGGATGCGTTCCATTAGATAGTAGGCGGGGTAACGGCCCACCTAGTCAACGATGGATAGGGGTTCTGAGAGGAAGGTCCCCCACATTGGAACTGAGACACGGTCCAAACTCCTACGGGGAGGCAGCAGTGAGGAATATTGGTCAATGGGCGATGGCCTGAACCAGCCAAGTAGCGTGAAGGATGACTGCCCTATGGGTTGTAAACTTCTTTTATAAAGGAATAAAGTCGGGTATGCATACCCGTTTGCATGTACTTTATGAATAA | *Bacteroides dorei* HS2_L_2_B_045b |
| **B20** | ACGCTAGCTACAGGCTTAACACATGCAAGTCGAGGGGCAGCATGGTCTTAGCTTGCTAAGGCTGATGGCGACCGGCGCACGGGTGAGTAACACGTATCCAACCTGCCGTCTACTCTTGGCCAGCCTTCTGAAAGGAAGATTAATCCAGGATGGGATCATGAGTTCACATGTCCGCATGATTAAAGGTATTTTCCGGTAGACGATGGGGATGCGTTCCATTAGATAGTAGGCGGGGTAACGGCCCACCTAGTCAACGATGGATAGGGGTTCTGAGAGGAAGGTCCCCCACATTGGAACTGAGACACGGTCCAAACTCCTACGGGGAGGCAGCAGTGAGGAATATTGGTCAATGGGCGATGGCCTGAACCAGCCAAGTAGCGTGAAGGATGACTGCCCTATGGGTTGTAAACTTCTTTTATAAAGGATAAAGTCGGGTATGCATACCCGTTTGCATGTACTTTATGAATAA | *Bacteroides dorei* HS2_L_2_B_045b |
| **B21** | ACGCTAGCTACAGGCTTAACACATGCAAGTCGAGGGGCAGCATGGTCTTAGCTTGCTAAGGCCGATGGCGACCGGCGCACGGGTGAGTAACACGTATCCAACCTGCCGTCTACTCTTGGACAGCCTTCTGAAAGGAAGATTAATACAAGATGGCATCATGAGTCCGCATGTTCACATGATTAAAGGTATTCCGGTAGACGATGGGGATGCGTTCCATTAGATAGTAGGCGGGGTAACGGCCCACCTAGTCTTCGATGGATAGGGGTTCTGAGAGGAAGGTCCCCCACATTGGAACTGAGACACGGTCCAAACTCCTACGGGAGGCAGCAGTGAGGAATATTGGTCAATGGACGAGAGTCTGAACCAGCCAAGTAGCGTGAAGGATGACTGCCCTATGGGTTGTAAACTTCTTTTATACGGGAATAAAGTGAGGCACGTGTGCCTTTTTGTATGTACCGTATGAATAA | *Bacteroides vulgatus* ATTC 8482 |
| **B22** | ACGCTAGCTACAGGCTTAACACATGCAAGTCGAGGGGCAGCATGAACTTAGCTTGCTAAGTTTGATGGCGACCGGCGCACGGGTGAGTAACACGTATCCAACCTGCCGATGACTCGGGGATAGCCTTTCGAAAGAAAGATTAATACCCGATGGCATAGTTCTTCCGCATGGTAGAACTATTAAAGGATTTCGGTCATCGATGGGGATGCGTTCCATTAGGTTGTTGGCGGGGTAACGGCCCACCAAGCCTTCGATGGATAGGGGTTCTGAGAGGAAGGTCCCCCACATTGGAACTGAGACACGGTCCAAACTCCTACGGGAGGCAGCAGTGAGGAATATTGGTCAATGGACGAGAGTCTGAACCAGCCAAGTAGCGTGAAGGATGACTGCCCTATGGGTTGTAAACTTCTTTTATACGGGAATAAAGTGAGGCACGTGTGCCTTTTTGTATGTACCGTATGAATAA | *Bacteroides uniformis* JCM 5828 |
| **B23** | ACGCTAGCTACAGGCTTAACACATGCAAGTCGAGGGGCAGCATGGTCTTAGCTTGCTAAGGCCGATGGCGACCGGCGCACGGGTGAGTAACGCGTATCCAACCTGCCTTACACTCTTGGACAGCCTTCTGAAAGGGAGATTAATACAAGATGTTATCATGAGTAAGCATTTTCGCATGATTAAAGGTTTACCGGTGTAAGATGGGGATGCGTTCCATTAGATAGTAGGCGGGGTAACGGCCCACCTAGTCTTCGATGGATAGGGGTTCTGAGAGGAAGGTCCCCCACATTGGAACTGAGACACGGTCCAAACTCCTACGGGAGGCAGCAGTGAGGAATATTGGTCAATGGACGAGAGTCTGAACCAGCCAAGTAGCGTGAAGGATGAAGGTTCTATGGATTGTAAACTTCTTTTATACGGGAATAAACGAATCCACGTGTGGATTTTTGCATGTACCGTATGAATAA | *Bacteroides massiliensis* B84634 |
| **B24** | ACGCTAGCTACAGGCTTAACACATGCAAGTCGAGGGGCAGCATGAACTTAGCTTGCTAAGTTTGATGGCGACCGGCGCACGGGTGAGTAACACGTATCCAACCTGCCGATGACTCGGGGATAGCCTTTCGAAAGAAAGATTAATACCCGATGGCATAATTCTTCCGCATGGTAGAATTATTAAAGAATTTCGGTCATCGATGGGGATGCGTTCCATTAGGTTGTTGGCGGGGTAACGGCCCACCAAGCCTTCGATGGATAGGGGTTCTGAGAGGAAGGTCCCCCACATTGGAACTGAGACACGGTCCAAACTCCTACGGGAGGCAGCAGTGAGGAATATTGGTCAATGGACGAGAGTCTGAACCAGCCAAGTAGCGTGAAGGATGACTGCCCTATGGGTTGTAAACTTCTTTTATACGGGAATAAAGTGAGGCACGTGTGCCTTTTTGTATGTACCGTATGAATAA | *Bacteroides uniformis* JCM 5828 |
| **B25** | ACGCTAGCTACAGGCTTAACACATGCAAGTCGAGGGGCAGCATGAACTTAGCTTGCTAAGTTTGATGGCGACCGGCGCACGGGTGAGTAACACGTATCCAACCTGCCGATGACTCGGGGATAGCCTTTCGAAAGAAAGATTAATACCCGATGGCATAGTTCTTCCGCATGGTAGAACTATTAAAGAATTTCGGTCATCGATGGGGATGCGTTCCATTAGGTTGTTGGCGGGGTAACGGCCCACCAAGCCTTCGATGGATAGGGGTTCTGAGAGGAAGGTCCCCACATTGGAACTGAGACACGGTCCAAACTCCTACGGGAGGCAGCAGTGAGGAATATTGGTCAATGGACGAGAGTCTGAACCAGCCAAGTAGCGTGAAGGATGACTGCCCTATGGGTTGTAAACTTCTTTTATACGGGAATAAAGTGAGGCACGTGTGCCTTTTTGTATGTACCGTATGAATAA | *Bacteroides uniformis* JCM 5828 |
| **B26** | ACGCTAGCTACAGGCTTAACACATGCAAGTCGAGGGGCAGCATGAACTTAGCTTGCTAAGTTTGATGGCGACCGGCGCACGGGTGAGTAACACGTATCCAACCTTCCGTTTACTCAGGGATAGCCTTTCGAAAGAAAGATTAATACCTGATAGTATGGTGAGATTGCATGATAGCACCATTAAAGATTTATTGGTAAACGATGGGGATGCGTTCCATTAGGTAGTAGGCGGGGTAACGGCCCACCTAGCCTACGATGGATAGGGGTTCTGAGAGGAAGGTCCCCCACATTGGAACTGAGACACGGTCCAAACTCCTACGGGAGGCAGCAGTGAGGAATATTGGTCAATGGGCGAGAGCCTGAACCAGCCAAGTAGCGTGAAGGATGAAGGTCCTACGGATTGTAAACTTCTTTTATACGGGAATAAAGTATCCTACGTGTAGGATTTTGTATGTACCGTATGAATAA | *Bacteroides coprocola* M16 |
| **B27** | ACGCTAGCTACGGGCTTAACACATGCAAGTCGAGGGGCAGCATGGTCTTAGCTTGCTAAGGCTGATGGCGACCGGCGCACGGGTGAGTAACACGTATCCAACCTGCCGTCTACTCTTGGCCAGCCTTCTGAAAGGAAGATTAATCCAGGATGGCATCATGAGTTCACATGTCCGCATGATTAAAGGTATTTTCCGGTAGACGATGGGGATGCGTTCCATTAGATAGTAGGCGGGGTAACGGCCCACCTAGTCAACGATGGATAGGGGTTCTGAGAGGAAGGTCCCCCACATTGGAACTGAGACACGGTCCAAACTCCTACGGGAGGCAGCAGTGAGGAATATTGGTCAATGGGCGATGGCCTGAACCAGCCAAGTAGCGTGAAGGATGACTGCCCTATGGGTTGTAAACTTCTTTTATAAAGGAATAAAGTCGGGTATGCATACCCGTTTGCATGTACTTTATGAATAA | *Bacteroides coprocola* 175 |
| **B28** | ACGCTAGCTACAGGCTTAACACATGCAAGTCGAGGGGCAGCATGGTCTTAGCTTGCTAAGGCCGATGGCGACCGGCGCACGGGTGAGTAACACGTATCCAACCTGCCGTCTACTCTTGGACAGCCTTCTGAAAGGAAGATTAATACAAGATGGCATCATGAGTTCACATGTTCACATGATTAAAGGTATTCCGGTAGACGATGGGGATGCGTTCCATTAGATAGTAGGCGGGGTAACGGCCCACCTAGTCTTCGATGGATAGGGGTTCTGAGAGGAAGGTCCCCCACATTGGAACTGAGACACGGTCCAAACTCCTACGGGGAGGCAGCAGTGAGGAATATTGGTCAATGGGCGCAGGCCTGAACCAGCCAAGTAGCGTGAAGGATGACTGCCCTATGGGTTGTAAACTTCTTTTATAAAGGAATAAAGTCGGGTATGTATACCCGTTTGCATGTACTTTATGAATAA | *Bacteroides vulgatus* ATCC 8482 |
| **B29** | ACGCTAGCTACAGGCTTAACACATGCAAGTCGAGGGGCAGCATGGTCTTAGCTTGCTAAGGCCGATGGCGACCGGCGCACGGGTGAGTAACACGTATCCAACCTGCCGTCTACTCTTGGACAGCCTTCTGAAAGGAAGATTAATACAAGATGGCATCATGAGTCCACATGTTCACATGATTAAAGGTATTCCGGTAGACGATGGGGATGCGTTCCATTAGATAGTAGGCGGGGTAACGGCCCACCTAGTCTTCGATGGATAGGGGTTCTGAGAGGAAGGTCCCCCACATTGGAACTGAGACACGGTCCAAACTCCTACGGGGAGGCAGCAGTGAGGAATATTGGTCAATGGGCGAGAGCCTGAACCAGCCAAGTAGCGTGAAGGATGACTGCCCTATGGGTTGTAAACTTCTTTTATAAAGGATAAAGTCGGGTATGCATACCCGTTTGCATGTACTTTATGAATAA | *Bacteroides vulgatus* ATCC 8482 |
| **B30** | ACGCTAGCTACAGGCTTAACACATGCAAGTCGAGGGGCAGCGGGATTGAAGCTTGCTTCAATTGCCGGCGACCGGCGCACGGGTGAGTAACGCGTATCCAACCTTCCGTACACTCAGGGATAGCCTTTCGAAAGAAAGATTAATACCTGATGGTATCTTAAGCACACATGTAATTAAGATTAAAGATTTATCGGTGTACGATGGGGATGCGTTCCATTAGGTAGTAGGCGGGGTAACGGCCCACCTAGCCAACGATGGATAGGGGTTCTGAGAGGAAGGTCCCCCACATTGGAACTGAGACACGGTCCAAACTCCTACGGGAGGCAGCAGTGAGGAATATTGGTCAATGGACGAGAGTCTGAACCAGCCAAGTAGCGTGAAGGATGAAGGTCCTACGGATTGTAAACTTCTTTTATAAGGGAATAAACCCTCCCACGTGTGGGAGCTTGTATGTACCTTATGAATAA | *Bacteroides plebeius* M12 |
| **B31** | ACGCTAGCTACAGGCTTAACACATGCAAGTCGAGGGGCAGCATGAACTTAGCTTGCTAAGTTTGATGGCGACCGGCGCACGGGTGAGTAACACGTATCCAACCTTCCGTTTACTCAGGGATAGCCTTTCGAAAGAAAGATTAATACCTGATAGTATGGTGAGATTGCATGATAACACCATTAAAGATTTATTGGTAAACGATGGGGATGCGTTCCATTAGGTAGTAGGCGGGGTAACGGCCCACCTAGCCTGCGATGGATAGGGGTTCTGAGAGGAAGGTCCCCCACATTGGAACTGAGACACGGTCCAAACTCCTACGGGAGGCAGCAGTGAGGAATATTGGTCAATGGGCGAGAGCCTGAACCAGCCAAGTAGCGTGAAGGATGAAGGTCCTACGGATTGTAAACTTCTTTTATACGGGAATAAAGTTTCCTACGTGTAGGATTTTGTATGTACCGTATGAATAA | *Bacteroides coprocola* M16 |
| **B32** | ACGCTAGCTACAGGCTTAACACATGCAAGTCGAGGGGCAGCATGATTGAAGCTTGCTTCAATCGATGGCGACCGGCGCACGGGTGAGTAACACGTATCCAACCTGCCGATAACTCGGGGATAGCCTTTCGAAAGAAAGATTAATACCCGATAGTATAGTATTTCCGCATGGTTTCACTATTAAAGAATTTCGGTTATCGATGGGGATGCGTTCCATTAGATAGTTGGCGGGGTAACGGCCCACCAAGTCAACGATGGATAGGGGTTCTGAGAGGAAGGTCCCCCACATTGGAACTGAGACACGGTCCAAACTCCTACGGGAGGCAGCAGTGAGGAATATTGGTCAATGGACGAGAGTCTGAACCAGCCAAGTAGCGTGAAGGATGACTGCCCTATGGGTTGTAAACTTCTTTTATACGGGAATAAAGTGGAGTATGCATACTCCTTTGTATGTACCGTATGAATAA | *Bacteroides egghertii* JCM 12986 |
| **B33** | ACGCTAGCTACAGGCTTAACACATGCAAGTCGAGGGGCAGCATGGTCTTAGCTTGCTAAGGCCGATGGCGACCGGCGCACGGGTGAGTAACGCGTATCCAACCTGCCTTACACTCTTGGACAGCCTTCTGAAAGGGAGATTAATACAAGATGTTATCATGAGTAAGCATTTTCGCATGATTAAAGGTTTACCGGTGTAAGATGGGGATGCGTTCCATTAGATAGTAGGCGGGGTAACGGCCCACCTAGTCTTCGATGGATAGGGGTTCTGAGAGGAAGGTCCCCCCACATTGGAACTGAGACACGGTCCAAACTCCTACGGGAGGCAGCAGTGAGGAATATTGGTCAATGGACGAGAGTCTGAACCAGCCAAGTAGCGTGAAGGATGAAGGTTCTATGGATTGTAAACTTCTTTTATACGGGAATAAACGAATCCACGTGTGGATTTTTGCATGTACCGTATGAATAA | *Bacteroides massiliensis* B84634 |
| **B34** | ACGCTAGCTACAGGCTTAACACATGCAAGTCGAGGGGCAGCATGGTCTTAGCTTGCTAAGGCTGATGGCGACCGGCGCACGGGTGAGTAACACGTATCCAACCTGCCGTCTACTCTTGGCCAGCCTTCTGAAAGGAAGATTAATCCAGGATGGCATCATGAGTTCACATGTCCGCATGATTAAAGGTATTTTCCGGTAGACGATGGGGATGCGTTCCATTAGATAGTAGGCGGGGTAACGGCCCACCTAGTCAACGATGGATAGGGGTTCTGAGAGGAAGGTCCCCCACATTGGAACTGAGACACGGTCCAAACTCCTACGGGAGGCAGCAGTGAGGAATATTGGTCAATGGGCGATGGCCTGAACCAGCCAAGTAGCGTGAAGGATGACTGCCCTATGGGTTGTAAACTTCTTTTATAAAGGATAAAGTCGGGTATGCATACCCGTTTGCATGTACTTTATGAATAA | *Bacteroides dorei* 175 |
| **B35** | ACGCTAGCTACAGGCTTAACACATGCAAGTCGAGGGGCAGCATGGTCTTAGCTTGCTAAGGCCGATGGCGACCGGCGCACGGGTGAGTAACACGTATCCAACCTGCCGTCTACTCTTGGACAGCCTTCTGAAAGGAAGATTAATACAAGATGGCATCATGAGTTCACATGTTCACATGATTAAAGGTATTCCGGTAGACGATGGGGATGCGTTCCATTAGATAGTAGGCGGGGTAACGGCCCACCTAGTCTTCGATGGATAGGGGTTCTGAGAGGAAGGTCCCCCCACATTGGAACTGAGACACGGTCCAAACTCCTACGGGAGGCAGCAGTGAGGAATATTGGTCAATGGGCGCAGGCCTGAACCAGCCAAGTAGCGTGAAGGATGACTGCCCTATGGGTTGTAAACTTCTTTTATAAAGGAATAAAGTCGGGTATGTATACCCGTTTGCATGTACTTTATGAATAA | *Bacteroides vulgatus* ATCC 8482 |
| **B36** | ACGCTAGCTACAGGCTTAACACATGCAAGTCGAGGGGCAGCATGGTCTTAGCTTGCTAAGGCCGATGGCGACCGGCGCACGGGTGAGTAACACGTATCCAACCTGCCGTCTACTCTTGGACAGCCTTCTGAAAGGAAGATTAATACAAGATGGCATCATGAGTCCGCATGTTCACATGATTAAAGGTATTCCGGTAGACGATGGGGATGCGTTCCATTAGATAGTAGGCGGGGTAACGGCCCACCTAGTCTTCGATGGATAGGGGTTCTGAGAGGAAGGTCCCCCACATTGGAACTGAGACACGGTCCAAACTCCTACGGGAGGCAGCAGTGAGGAATATTGGTCAATGGGCGAGAGCCTGAACCAGCCAAGTAGCGTGAAGGATGACTGCCCTATGGGTTGTAAACTTCTTTTATACGGGAATAAAGTGAGGCACGTGTGCCTTTTTGTATGTACCGTATGAATAA | *Bacteroides vulgatus* ATCC 8482 |
| **B37** | ACGCTAGCTACAGGCTTAACACATGCAAGTCGAGGGGCAGCATGGTCTTAGCTTGCTAAGACTGATGGCGACCGGCGCACGGGTGAGTAACACGTATCCAACCTGCCGTCTACTCTTGGACAGCCTTCTGAAAGGAAGATTAATACAAGATGGCATCATGAGTCCGCATGTTCACATGATTAAAGGTATTCCGGTAGACGATGGGGATGCGTTCCATTAGATAGTAGGCGGGGTAACGGCCCACCTAGTCTTCGATGGATAGGGGTTCTGAGAGGAAGGTCCCCCCACATTGGAACTGAGACACGGTCCAAACTCCTACGGGAGGCAGCAGTGAGGAATATTGGTCAATGGGCGAGAGCCTGAACCAGCCAAGTAGCGTGAAGGATGACTGCCCTATGGGTTGTAAACTTCTTTTATAAAGGATAAAGTCGGGTATGGATACCCGTTTGCATGTACTTTATGAATAA | *Bacteroides vulgatus* ATCC 8482 |
| **B38** | ACGCTAGCTACAGGCTTAACACATGCAAGTCGAGGGGCAGCATGGTCTTAGCTTGCTAAGACTGATGGCGACCGGCGCACGGGTGAGTAACACGTATCCAACCTGCCGTCTACTCTTGGACAGCCTTCTGAAAGGAAGATTAATACAAGATGGCATCATGAGTCCGCATGTTCACATGATTAAAGGTATTCCGGTAGACGATGGGGATGCGTTCCATTAGATAGTAGGCGGGGTAACGGCCCACCTAGTCTTCGATGGATAGGGGTTCTGAGAGGAAGGTCCCCCACATTGGAACTGAGACACGGTCCAAACTCCTACGGGAGCAGCAGTGAGGAATATTGGTCAATGGGCGAGAGCCTGAACCAGCCAAGTAGCGTGAAGGATGACTGCCCTATGGGTTGTAAACTTCTTTTATAAAGGATAAAGTCGGGTATGGATACCCGTTTGCATGTACTTTATGAATAA | *Bacteroides vulgatus* ATCC 8482 |
| **B39** | ACGCTAGCTACAGGCTTAACACATGCAAGTCGAGGGGCAGCATGGTCTTAGCTTGCTAAGGCTGATGGCGACCGGCGCACGGGTGAGTAACACGTATCCAACCTGCCGTCTACTCTTGGCCAGCCTTCTGAAAGGAAGATTAATCCAGGATGGGGATCATGAGTTCACATGTCCGCATGATTAAAGGTATTTTCCGGTAGACGATGGGGATGCGTTCCATTAGATAGTAGGCGGGGTAACGGCCCACCTAGTCAACGATGGATAGGGGTTCTGAGAGGAAGGTCCCCCACATTGGAACTGAGACACGGTCCAAACTCCTACGGGAGGCAGCAGTGAGGAATATTGGTCAATGGGCGATGGCCTGAACCAGCCAAGTAGCGTGAAGGATGACTGCCCTATGGGTTGTAAACTTCTTTTATAAAGGATAAAGTCGGGTATGCATACCCGTTTGCATGTACTTTATGAATAA | *Bacteroides dorei* 175 |
| **B40** | ACGCTAGCTACAGGCTTAACACATGCAAGTCGAGGGGCAGCATGAACTTAGCTTGCTAAGTTTGATGGCGACCGGCGCACGGGTGAGTAACACGTATCCAACCTGCCGATGACTCGGGGATAGCCTTTCGAAAGAAAGATTAATACCCGATGGCATAATTCTTCCGCATGGTAGAACTATTAAAGAATTTCGGTCATCGATGGGGATGCGTTCCATTAGGTTGTTGGCGGGGTAACGGCCCACCAAGCCTTCGATGGATAGGGGTTCTGAGAGGAAGGTCCCCCACATTGGAACTGAGACACGGTCCAAACTCCTACGGGGAGGCAGCAGTGAGGAATATTGGTCAATGGACGAGAGTCTGAACCAGCCAAGTAGCGTGAAGGATGACTGCCCTATGGGTTGTAAACTTCTTTTATACGGGAATAAAGTGAGGCACGTGTGCCTTTTTGTATGTACCGTATGAATAA | *Bacteroides uniformis* JCM 5828 |
| **B41** | ACGCTAGCTACAGGCTTAACACATGCAAGTCGAGGGGCAGCATGACCTAGCAATAGGTTGATGGCGACCGGCGCACGGGTGAGTAACACGTATCCAACCTGCCGATTATTCCGGGATAGCCTTTCGAAAGAAAGATTAATACTGGATAGCATAACGAGAAGGCATCTTCTTGTTATTAAAGAATTTCGATAATCGATGGGGATGCGTTCCATTAGTTTGTTGGCGGGGTAACGGCCCACCAAGACATCGATGGATAGGGGTTCTGAGAGGAAGGTCCCCCACATTGGAACTGAGACACGGTCCAAACTCCTACGGGAGGCAGCAGTGAGGAATATTGGTCAATGGACGAGAGTCTGAACCAGCCAAGTAGCGTGAAGGATGACTGCCCTATGGGTTGTAAACTTCTTTTATATGGGAATAAAGTGAGCCACGTGTGGCTTTTTGTATGTACCATACGAATAA | *Bacteroides intestinalis* 341 |
| **B42** | ACGCTAGCTACAGGCTTAACACATGCAAGTCGAGGGGCAGCATGAACTTAGCTTGCTAAGTTTGATGGCGACCGGCGCACGGGTGAGTAACACGTATCCAACCTGCCGATGACTCGGGGATAGCCTTTCGAAAGAAAGATTAATACCCGATGGCATAGTTCTTCCGCATGGTGGAACTATTAAAGAATTTCGGTCATCGATGGGGATGCGTTCCATTAGGTTGTTGGCGGGGTAACGGCCCACCAAGCCTTCGATGGATAGGGGTTCTGAGAGGAAGGTCCCCCACATTGGAACTGAGACACGGTCCAAACTCCTACGGGAGGCAGCAGTGAGGAATATTGGTCAATGGACGAGAGTCTGAACCAGCCAAGTAGCGTGAAGGATGACTGCCCTATGGGTTGTAAACTTCTTTTATACGGGAATAAAGTGAGGCACGCGTGCCTTTTTGTATGTACCGTATGAATAA | *Bacteroides uniformis* JCM 5828 |
| **B43** | ACGCTAGCTACAGGCTTAACACATGCAAGTCGAGGGGCAGCATGAACTTAGCTTGCTAAGTTTGATGGCGACCGGCGCACGGGTGAGTAACACGTATCCAACCTGCCGATGACTCGGGGATAGCCTTTCGAAAGAAAGATTAATACCCGATGGCATAGTTCTTCCGCATGGTAGAACTATTAAAGAATTTCGGTCATCGATGGGGATGCGTTCCATTAGGTTGTTGGCGGGGTAACGGCCCACCAAGCCTTCGATGGATAGGGGTTCTGAGAGGAAGGTCCCCCACATTGGAACTGAGACACGGTCCAAACTCCTACGGGGAGGCAGCAGTGAGGAATATTGGTCAATGGACGAGAGTCTGAACCAGCCAAGTAGCGTGAAGGATGACTGCCCTATGGGTTGTAAACTTCTTTTATACGGGAATAAAGTGAGGCACGCGTGCCTTTTTGTATGTACCGTATGAATAA | *Bacteroides uniformis* JCM 5828 |
| **B44** | ACGCTAGCTACAGGCTTAACACATGCAAGTCGAGGGGCAGCATGATTGAAGCTTGCTTCAATTGATGGCGACCGGCGCACGGGTGAGTAACACGTATCCAACCTGCCGATAACTCGGGGATAGCCTTTCGAAAGAAAGATTAATACCCGATAGTATAGTATTTCCGCATGGTTTCACTATTAAAGAATTTCGGTTATCGATGGGGATGCGTTCCATTAGATAGTTGGCGGGGTAACGGCCCACCAAGTCAACGATGGATAGGGGTTCTGAGAGGAAGGTCCCCCACATTGGAACTGAGACACGGTCCAAACTCCTACGGGAGGCAGCAGTGAGGAATATTGGTCAATGGACGAGAGTCTGAACCAGCCAAGTAGCGTGAAGGATGACTGCCCTATGGGTTGTAAACTTCTTTTATACGGGAATAAAGTGGAGTATGCATACTCCTTTGTATGTACCGTATGAATAA | *Bacteroides eggerthii* JCM 12986 |
| **B45** | ACGCTAGCTACAGGCTTAACACATGCAAGTCGAGGGGCAGCATTTCAGTTTGCTTGCAAACTGGAGATGGCGACCGGCGCACGGGTGAGTAACACGTATCCAACCTGCCGATAACTCGGGGATAGCCTTTCGAAAGAAAGATTAATACCCGATGGTATAATCAGACCGCATGGTCTTGTTATTAAAGAATTTCGGTTATCGATGGGGATGCGTTCCATTAGGCAGTTGGTGAGGTAACGGCTCACCAAACCTTCGATGGATAGGGGTTCTGAGAGGAAGGTCCCCCACATTGGAACTGAGACACGGTCCAAACTCCTACGGGAGGCAGCAGTGAGGAATATTGGTCAATGGGCGCAGGCCTGAACCAGCCAAGTAGCGTGAAGGATGACTGCCCTATGGGTTGTAAACTTCTTTTATATGGGAATAAAGTTTTCCACGTGTGGAATTTTGTATGTACCATATGAATAA | *Bacteroides thetaiotaomicron* VPI-5482 |
| **B46** | ACGCTAGCTACAGGCTTAACACATGCAAGTCGAGGGGCAGCATGGTCTTAGCTTGCTAAGGCCGATGGCGACCGGCGCACGGGTGAGTAACACGTATCCAACCTGCCGTCTACTCTTGGACAGCCTTCTGAAAGGAAGATTAATACAAGATGGCATCATGAGTTCACATGTTCACATGATTAAAGGTATTCCGGTAGACGATGGGGATGCGTTCCATTAGATAGTAGGCGGGGTAACGGCCCACCTAGTCTTCGATGGATAGGGGTTCTGAGAGGAAGGTCCCCCCACATTGGAACTGAGACACGGTCCAAACTCCTACGGGGAGGCAGCAGTGAGGAATATTGGTCAATGGGCGCAGGCCTGAACCAGCCAAGTAGCGTGAAGGATGACTGCCCTATGGGTTGTAAACTTCTTTTATAAAGGAATAAAGTCGGGTATGTATACCCGTTTGCATGTACTTTATGAATAA | *Bacteroides vulgatus* ATCC 8482 |
| **B47** | ACGCTAGCTACAGGCTTAACACATGCAAGTCGAGGGGCAGCATTTTAGTTTGCTTGCAAACTGAAGATGGCGACCGGCGCACGGGTGAGTAACACGTATCCAACCTGCCGATAACTCCGGAATAGCCTTTCGAAAGAAAGATTAATACCGGATAGCATACGAATATCGCATGATATTTTTATTAAAGAATTTCGGTTATCGATGGGGATGCGTTCCATTAGTTTGTTGGCGGGGTAACGGCCCACCAAGACTACGATGGATAGGGGTTCTGAGAGGAAGGTCCCCCACATTGGAACTGAGACACGGTCCAAACTCCTACGGGAGGCAGCAGTGAGGAATATTGGTCAATGGGCGAGAGCCTGAACCAGCCAAGTAGCGTGAAGGATGAAGGCTCTATGGGTCGTAAACTTCTTTTATATGGGAATAAAGTTTTCCACGTGTGGAATTTTGTATGTACCATATGAATAA | *Bacteroides ovatus* JCM 5824 |
| **B48** | ACGCTAGCTACAGGCTTAACACATGCAAGTCGAGGGGCAGCATGAACTTAGCTTGCTAAGTTTGATGGCGACCGGCGCACGGGTGAGTAACACGTATCCAACCTTCCGTTTACTCAGGGATAGCCTTTCGAAAGAAAGATTAATACCTGATAGTATGGTGAGATTGCATGATAGCACCATTAAAGATTTATTGGTAAACGATGGGGATGCGTTCCATTAGGTAGTAGGCGGGGTAACGGCCCACCTAGCCGACGATGGATAGGGGTTCTGAGAGGAAGGTCCCCCACATTGGAACTGAGACACGGTCCAAACTCCTACGGGGAGGCAGCAGTGAGGAATATTGGTCAATGGGCGAGAGCCTGAACCAGCCAAGTAGCGTGAAGGATGAAGGTCCTACGGATTGTAAACTTCTTTTATACGGGAATAAAGTATCCTACGTGTAGGATTTTGTATGTACCGTATGAATAA | *Bacteroides coprocola* M16 |
| **B49** | ACGCTAGCTACAGGCTTAACACATGCAAGTCGAGGGGCAGCATGAACTTAGCTTGCTAAGTTTGATGGCGACCGGCGCACGGGTGAGTAACACGTATCCAACCTGCCGATGACTCGGGGATAGCCTTTCGAAAGAAAGATTAATACCCGATGGCATAGTTCTTCCGCATGGTGGAACTATTAAAGAATTTCGGTCATCGATGGGGATGCGTTCCATTAGGTTGTTGGCGGGGTAACGGCCCACCAAGCCTTCGATGGATAGGGGTTCTGAGAGGAAGGTCCCCACATTGGAACTGAGACACGGTCCAAACTCCTACGGGAGGCAGCAGTGAGGAATATTGGTCAATGGACGAGAGTCTGAACCAGCCAAGTAGCGTGAAGGATGACTGCCCTATGGGTTGTAAACTTCTTTTATACGGGAATAAAGTGAGGCACGTGTGCCTTTTTGTATGTACCGTATGAATAA | *Bacteroides uniformis* JCM 5828 |
| **B50** | ACGCTAGCTACAGGCTTAACACATGCAAGTCGAGGGGCAGCATGAACTTAGCTTGCTAAGTTTGATGGCGACCGGCGCACGGGTGAGTAACACGTATCCAACCTGCCGATGACTCGGGGATAGCCTTTCGAAAGAAAGATTAATACCCGATGGCATAGTTCTTCCGCATGGTGGAACTATTAAAGAATTTCGGTCATCGATGGGGATGCGTTCCATTAGGTTGTTGGCGGGGTAACGGCCCACCAAGCCTTCGATGGATAGGGGTTCTGAGAGGAAGGTCCCCCACATTGGAACTGAGACACGGTCCAAACTCCTACGGGGAGGCAGCAGTGAGGAATATTGGTCAATGGACGAGAGTCTGAACCAGCCAAGTAGCGTGAAGGATGACTGCCCTATGGGTTGTAAACTTCTTTTATACGGGAATAAAGTGAGGCACGTGTGCCTTTTTGTATGTACCGTATGAATAA | *Bacteroides uniformis* JCM 5828 |
| **B51** | ACGCTAGCTACAGGCTTAACACATGCAAGTCGAGGGGCAGCATGAACTTAGCTTGCTAAGTTTGATGGCGACCGGCGCACGGGTGAGTAACACGTATCCAACCTGCCGATGACTCGGGGATAGCCTTTCGAAAGAAAGATTAATACCCGATGGCATAGTTCTTCCGCATGGTAGAACTATTAAAGAATTTCGGTCATCGATGGGGATGCGTTCCATTAGGTTGTTGGCGGGGTAACGGCCCACCAAGCCTTCGATGGATAGGGGTTCTGAGAGGAAGGTCCCCCACATTGGAACTGAGACACGGTCCAAACTCCTACGGGAGCAGCAGTGAGGAATATTGGTCAATGGACGAGAGTCTGAACCAGCCAAGTAGCGTGAAGGATGACTGCCCTATGGGTTGTAAACTTCTTTTATACGGGAATAAAGTGAGGCACGTGTGCCTTTTTGTATGTACCGTATGAATAA | *Bacteroides uniformis* JCM 5828 |

**Supplementary Figure Legends**

**Supplementary Figure 1.** Bar plot of *Prevotella* (A) and *Bacteroides* (B) genera relative abundance ordered by size and line chart showing the number of different oligotypes identified in the same subject.

**Supplementary Figure 2.** Pie charts showing the average relative abundance of *Prevotella* (A) and *Bacteroides* (B) oligotypes in omnivores (left side) and non-omnivores (right side) subjects. For clarity, only *Bacteroides* oligotypes showing > 1% abundance in at least 10% of the subjects in each group are shown.

**Supplementary Figure 3.** Correlation between *Prevotella* (A) and *Bacteroides* (B) oligotypes and metabolome. Heatplot showing Spearman’s correlations between oligotypes, urinary TMAO and faecal SCFA levels. Rows and columns are clustered by Euclidean distance and Ward linkage hierarchical clustering. The intensity of the colors represents the degree of association between oligotypes and metabolites as measured by the Spearman’s correlations. Asterisks denote significant correlations after p-value corrections (P<0.05).

**Supplementary Figure 4.** Heatplot showing the percent nucleotide identity between each pair of oligotypes within *Prevotella* (A) and *Bacteroides* (B) genera. Each oligotype is identified with the best match found in NCBI nr database, with the percent of query coverage and the percent of identity. Row and columns are clustered by Horn distance and Ward linkage hierarchical clustering.

**Supplementary Figure 5.** Significant co-occurrence and co-exclusion relationships between *Prevotella* (A) and *Bacteroides* (B) oligotypes. Strong correlations are indicated by large circles, whereas weak correlations are indicated by small circles. The colors of the scale bar denote the nature of the correlation, with 1 indicating a perfectly positive correlation (dark blue) and -1 indicating a perfectly negative correlation (dark red) between two oligotypes. Only significant correlations (P<0.05) are shown.
